# Coordination of lignin and pectin remodeling by a NAC transcription factor regulates seed abscission in sesame (*Sesamum indicum* L.)

**DOI:** 10.1093/plphys/kiag762

**Published:** 2026-10-14

**Authors:** Eunsoo Lee, Jinsu Lee, Sungup Kim, Jeongeun Lee, Minsoo Han, Woo-Taek Jeon, Minsu Park, Myoung Hee Lee, Jungin Kim, Sangwoo Kim, Yeon Ju An, Eunyoung Oh, Min Young Kim, Jeong-Dong Lee, Chanseok Shin, Yuree Lee, Kwang-Soo Cho

**Affiliations:** Upland Crop Breeding Division, Department of Upland Crop Sciences, National Institute of Crop and Food Science, RDA, Miryang 50424, Republic of Korea; Research Institute of Basic Sciences, Seoul National University, Seoul 08826, Republic of Korea; Upland Crop Breeding Division, Department of Upland Crop Sciences, National Institute of Crop and Food Science, RDA, Miryang 50424, Republic of Korea; Upland Crop Breeding Division, Department of Upland Crop Sciences, National Institute of Crop and Food Science, RDA, Miryang 50424, Republic of Korea; School of Biological Sciences, Seoul National University, Seoul 08826, Republic of Korea; School of Biological Sciences, Seoul National University, Seoul 08826, Republic of Korea; Research Center for Plant Plasticity, Seoul National University, Seoul 08826, Republic of Korea; Plant Genomics and Breeding Institute, Seoul National University, Seoul 08826, Republic of Korea; Department of Agricultural Biotechnology, Seoul National University, Seoul 08826, Republic of Korea; Research Institute of Agriculture and Life Sciences, Seoul National University, Seoul 08826, Republic of Korea; Upland Crop Breeding Division, Department of Upland Crop Sciences, National Institute of Crop and Food Science, RDA, Miryang 50424, Republic of Korea; Upland Crop Breeding Division, Department of Upland Crop Sciences, National Institute of Crop and Food Science, RDA, Miryang 50424, Republic of Korea; Upland Crop Breeding Division, Department of Upland Crop Sciences, National Institute of Crop and Food Science, RDA, Miryang 50424, Republic of Korea; Upland Crop Breeding Division, Department of Upland Crop Sciences, National Institute of Crop and Food Science, RDA, Miryang 50424, Republic of Korea; Planning and Coordination Division, National Institute of Crop and Food Science, RDA, Wanju 55365, Republic of Korea; Food Tech Resources Research Division, Department of Food Sciences, National Institute of Crop and Food Science, RDA, Jeonju 55365, Republic of Korea; School of Applied Biosciences, Kyungpook National University, Daegu 41566, Republic of Korea; Research Center for Plant Plasticity, Seoul National University, Seoul 08826, Republic of Korea; Plant Genomics and Breeding Institute, Seoul National University, Seoul 08826, Republic of Korea; Department of Agricultural Biotechnology, Seoul National University, Seoul 08826, Republic of Korea; Research Institute of Agriculture and Life Sciences, Seoul National University, Seoul 08826, Republic of Korea; School of Biological Sciences, Seoul National University, Seoul 08826, Republic of Korea; Research Center for Plant Plasticity, Seoul National University, Seoul 08826, Republic of Korea; Plant Genomics and Breeding Institute, Seoul National University, Seoul 08826, Republic of Korea; Highland Agriculture Research Institute, National Institute of Crop and Food Science, RDA, Pyeongchang 25342, Republic of Korea

## Abstract

Seed shattering, which facilitates seed dispersal, is essential for plant reproduction but causes substantial yield loss in sesame (*Sesamum indicum* L.). It occurs through capsule dehiscence and/or seed abscission from the placenta, either simultaneously or independently depending on the species. In this study, we investigated cell wall-associated factors underlying delayed seed abscission in the sesame cultivar Suwon195 (SW195) relative to the easy-shattering cultivar IT030100. Histological and transcriptome analyses indicated that cell wall remodeling, particularly involving pectin and lignin, contributes to the delayed abscission phenotype of SW195. SW195 showed higher pectin methylesterase activity and stronger accumulation of low-methyl-esterified homogalacturonan, together with lower polygalacturonase-associated pectin turnover, than IT030100. These changes are consistent with a pectin-remodeling state that favors wall stabilization in the SW195 abscission zone. Abscission zone lignification was also weaker in SW195. Pharmacological inhibition of lignin biosynthesis altered pectin staining and reduced seed abscission in IT030100, whereas monolignol supplementation restored both phenotypes, supporting a functional association between lignification, pectin-related wall remodeling, and abscission. Quantitative trait loci (QTL) mapping and whole-genome resequencing identified a major locus on chromosome 9 encompassing a NAC transcription factor (*SiNAC; SIN_1025412*) and a pectin methylesterase inhibitor (*SiPMEI; SIN_1025380*). Transient overexpression of *SiNAC* enhanced lignin biosynthesis in sesame. Together, these findings indicate that coordinated lignin deposition and pectin remodeling are associated with seed abscission in sesame and identify candidate genetic components for breeding reduced-shattering cultivars.

## Introduction

Seed shattering facilitates seed dispersal, and therefore plays a vital role in plant reproduction and the maintenance of natural plant populations. However, in agriculture, it poses a significant challenge by causing yield loss during harvesting. To address this, selecting traits that suppress seed shattering has been a central objective of crop domestication. While this goal has been successfully achieved in major crops such as rice, wheat, and maize (Lin et al. 2012; Ishikawa et al. 2022; Emebiri and Hildebrand 2023), it remains unresolved in certain species, including sesame (*Sesamum indicum* L.).

Sesame, a member of the family Pedaliaceae, is a key oilseed crop that is widely used in food, pharmaceuticals, and cosmetics (Zhang et al. 2021; Ma et al. 2022a, 2022b). Sesame yield is influenced by various factors, including plant type, capsule number, seed number per capsule, seed size, and the degree of seed shattering. One major challenge in mechanized harvesting is that sesame capsules mature sequentially rather than simultaneously. As a result, early-maturing seeds often disperse before harvest, thereby causing significant yield loss. This highlights the importance of controlling seed shattering to improve harvest efficiency (Qureshi et al. 2022).

In sesame, seed shattering can be regulated by controlling either capsule dehiscence or the abscission of seeds from the placenta. Capsule indehiscence sesame cultivars have been shown to improve yield and facilitate the use of mechanized farming techniques (Tripathy et al. 2019). In addition, key regulators controlling this process in sesame, including SiCL1 and SiKANADI1, have been identified by previous studies (Zhang et al. 2018; Teboul et al. 2022). However, antishattering traits associated with capsule indehiscence remain challenging for automated harvesting machinery such as combine harvesters, since releasing seeds from closed capsules can be difficult. Consequently, there is a growing need to develop strategies capable of regulating seed shattering by suppressing seed abscission and allowing normal capsule dehiscence.

Abscission is a tightly regulated process that occurs in specialized regions known as abscission zones (AZs). These zones are established early during organogenesis and remain developmentally quiescent, exhibiting meristem-like characteristics with small size and dense cytoplasm (Patterson 2001). Upon activation, various cell wall-modifying enzymes are secreted, collectively triggering cell separation in the middle lamella of the AZ (Patterson 2001). Rich in pectic substances, the middle lamella is critical for maintaining intercellular adhesion; thus, its remodeling is critical during the abscission process. The coordinated action of enzymes—including β-galactosidases, polygalacturonases (PGs), pectin methyl esterases (PMEs), and pectin methyl esterase inhibitors (PMEIs)—facilitates cell wall loosening, ultimately enabling organ detachment.

Effective regulation of lignin deposition is also critical for proper abscission. In crops such as rice and tomato, in which the tissues surrounding the AZ undergo extensive lignification, suppressing lignin accumulation during AZ development is essential. Genetic studies have revealed that key domestication traits related to abscission have been shown to be associated with the repression of lignin synthesis (Mao et al. 2000; Konishi et al. 2006; Nakano et al. 2012; Yoon et al. 2014; Jiang et al. 2019; Ning et al. 2023). For instance, in rice, knockout or knockdown mutants of *SH11*, *SH5*, and *SSH1* exhibit higher rates of lignin deposition in panicles, resulting in production of nonshattering phenotypes (Yoon et al. 2014; Jiang et al. 2019; Ning et al. 2023). Similarly, in tomato, mutations in MADS-box genes such as *JOINTLESS* and *MACROCALYX* lead to excessive lignin deposition at inflorescence joints (Nakano et al. 2012; Mao et al. 2000). In contrast, in species like *Arabidopsis thaliana*, where tissue lignification surrounding the floral AZ is minimal, lignin plays a different role. Before abscission, lignin is deposited specifically within the AZ cells of the floral organ that is primed to detach. This localized lignin deposition acts as a structural brace that guides the activity of cell wall-degrading enzymes and ensures that cell wall breakdown occurs precisely within the designated cell layer (Lee et al. 2018). This mechanism appears to be conserved across different kinds of abscission events, including leaf petiole detachment. This conservation of function underscores the importance of lignification for modulating the efficiency and spatial precision of abscission.

Efforts to develop antishattering cultivars by suppressing seed abscission have also been pursued in sesame, especially in the elite line Suwon195 (SW195). Although SW195 undergoes capsules dehisce at maturity similar to standard cultivars, it shows strong seed attachment to the placenta, conferring high resistance to seed shattering (Kim et al. 2004). This trait not only minimizes postmaturity seed loss but is also linked to an increased number of capsules per plant and greater overall yield. However, whether this enhanced placental adhesion is causally related to the regulation of seed abscission remains unclear. Despite its agronomic value, the genetic and molecular mechanisms underlying this trait—and seed abscission in sesame more broadly—are still largely unknown. Addressing this knowledge gap is essential for both improving sesame cultivars and ensuring yield stability.

In this study, we investigated the cellular and physiological mechanisms underlying delayed seed abscission in SW195 by comparison with the shattering cultivar IT030100. Our findings indicate that altered pectin remodeling contributes to the strong placenta adhesion phenotype of SW195, as reflected by changes in pectin-modifying enzyme activity and pectin status in the AZ. We further found that AZ-specific lignification was reduced in SW195 and was functionally associated with pectin-related wall remodeling and seed abscission. QTL mapping and whole-genome resequencing identified a major locus on chromosome 9 containing a NAC transcription factor (*SiNAC; SIN_1025412*) and a pectin methylesterase inhibitor (*SiPMEI; SIN_1025380*) as candidate genes. Transient *SiNAC* overexpression enhanced lignin biosynthesis, while its binding to pectin-related promoter fragments suggests a potential role in regulating pectin-related genes. Together, these findings provide insight into cell wall-associated mechanisms underlying the SW195 phenotype and identify candidate genetic components for breeding reduced-shattering cultivars.

## Results

### Transcriptome analysis of seed abscission in sesame cultivars

To investigate the molecular mechanisms involved in seed abscission in sesame, we compared the seed shattering phenotype of the shattering cultivar IT030100 and the antishattering elite line SW195. In sesame grown under greenhouse conditions, both the capsule and seeds are mostly developed by 30 days after flowering (DAF) (Fig. 1a). Capsule yellowing begins around 80 DAF, and approximately 10 days later, seed abscission is initiated in conjunction with capsule opening. Finally, capsule and seed drying are completes by approximately 100 DAF. At this stage, the shattering cultivar IT030100 showed complete seed detachment, whereas the seeds of SW195 remained firmly attached to the placenta, even after drying (Fig. 1, b and c). Although SW195 undergoes normal capsule dehiscence, seed abscission does not occur, suggesting that regulatory mechanisms govern this process differently in the two cultivars.

**Figure 1:**
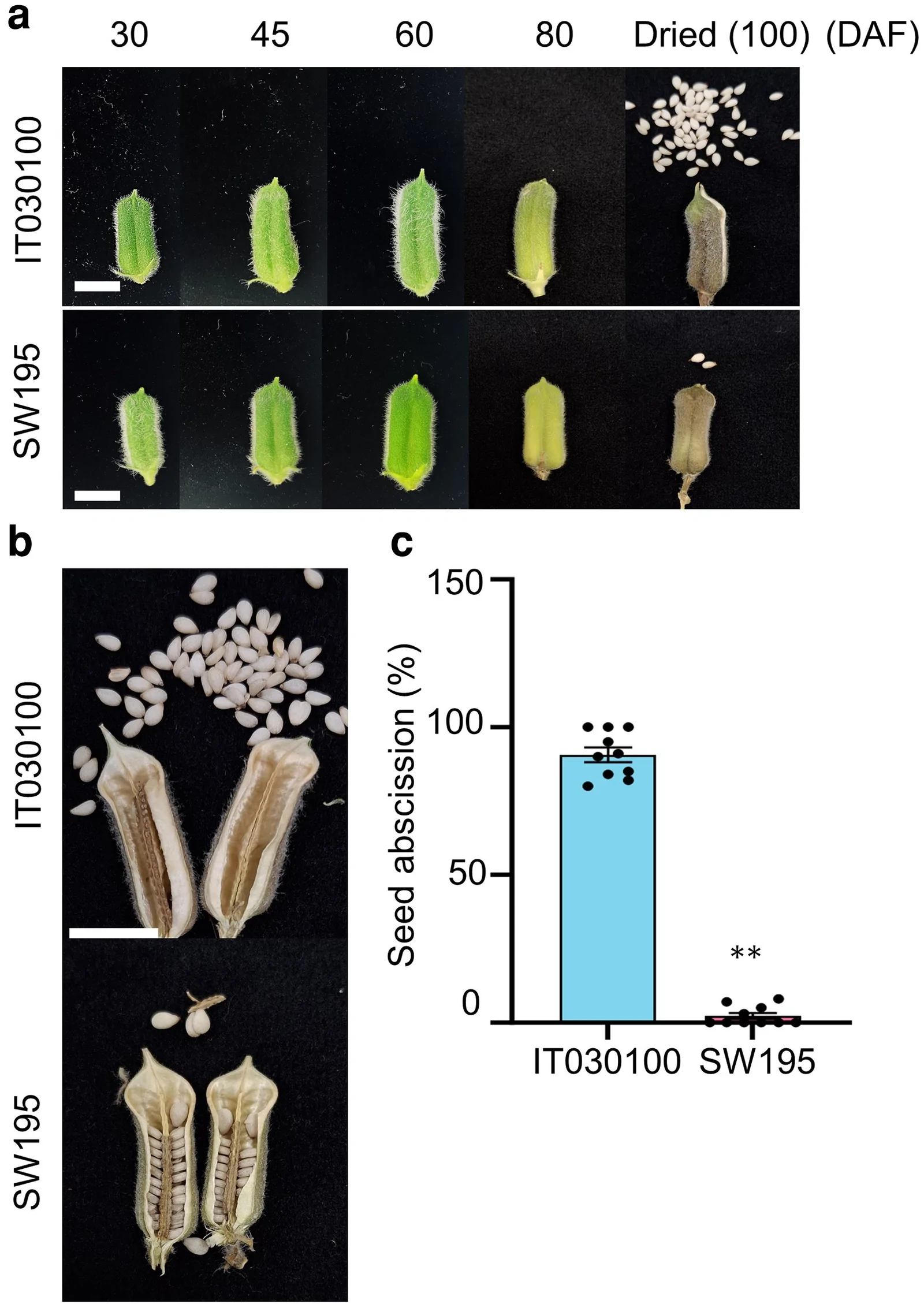
Seed abscission is obliterated in SW195 cultivar of *Sesamum indicum* L. a) Seed capsule and seed abscission phenotypes of IT030100 and SW195 plants arranged chronologically by timepoint (in DAF). b) Arrangement and seed abscission phenotype of seeds and opened capsule at 100 DAF. c) Quantification of seed abscission phenotypes of IT030100 and SW195 at 100 DAF. *n* = 10 capsules. All experiments were independently repeated at least three times. Error bars represent mean ± SD. **P < 0.01; Student's *t*-test (c). Bar indicates 1 cm (a, b).

To gain insight into the molecular basis of this phenotypic divergence, we performed a comparative transcriptome analysis of IT030100 and SW195. To pinpoint when key regulatory differences emerge during development, AZ tissues were manually isolated at 30, 45, and 60 DAF and subjected to RNA-seq at each time point (Fig. S1). Transcriptome comparisons revealed pronounced differences between the two lines at 30 and 60 DAF, with a more modest difference at 45 DAF (Fig. 2a and Table S1). These results suggest that key regulatory shifts occur predominantly during the early and late developmental stages.

**Figure 2:**
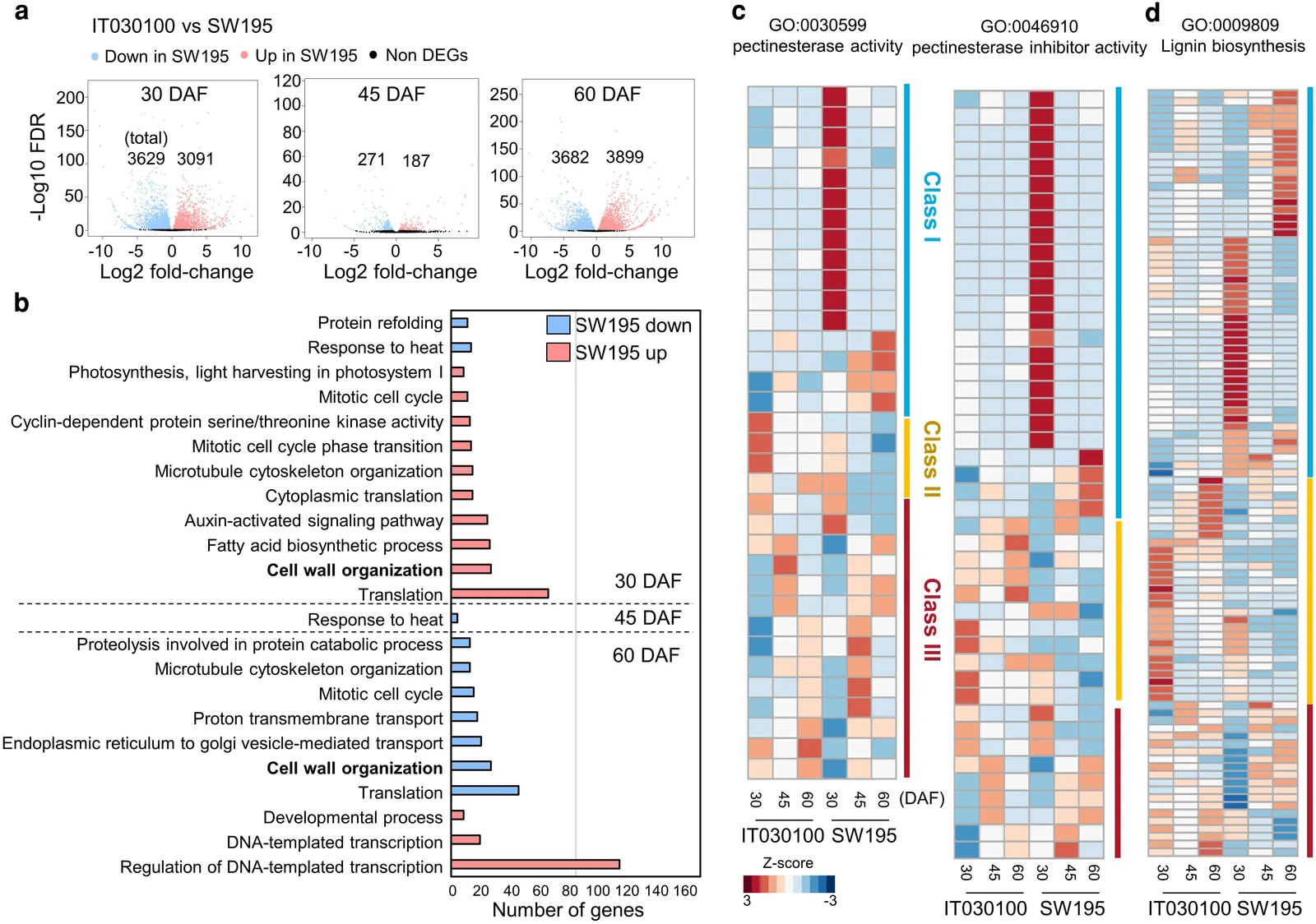
DEGs between IT030100 and SW195 sesame lines. a) Volcano plot depicting the log_2_-TMM for DEGs in SW195 relative to IT030100 at the indicated day after flowering (DAF). Genes were considered significantly differentially expressed based on a false discovery rate threshold of *P* < 0.05. See Fig. S1. b) GO term enrichment analysis. WEGO output for GO enrichment analysis for biological functions of DEGs with adjusted *P* < 0.05. See Table S2. c, d) Heatmap illustrating the expression profiles of genes associated with cell wall remodeling. Genes exhibiting a fold-change of ≥1.5 and a significance threshold of *P* < 0.05 were considered to be differentially expressed. See Tables S2–S5.

To better characterize these shifts, we performed gene ontology (GO) enrichment analysis on the differentially expressed genes (DEGs). We found significant enrichment of genes involved in cell wall organization, a critical biological process in abscission (Greenberg et al. 1975; Huberman and Goren 1979; Roberts et al. 2002; Lee et al. 2018) (Fig. 2b and Table S2). Further examination of these genes showed especially pronounced differences in the expression patterns of pectin modification-related gene families, including PME and PMEI (Fig. 2c, and Tables S3 and S4). In general, differences in gene expression clustered into three distinct classes: (i) genes exclusively expressed in SW195 (class I); (ii) genes expressed in both cultivars with altered expression levels in SW195 (class II); and (iii) genes showing shifts in the timing of expression between the two cultivars (class III) (Fig. 2c). Given their critical role in modulating pectin dynamics, the precise regulation of PME and PMEI genes likely contributes to proper seed abscission. Moreover, the diverse and complex expression patterns observed suggest that the regulatory divergence between IT030100 and SW195 involves more than a few simple changes in expression level or timing, and instead reflects the fact that a sophisticated regulatory network underlies AZ differentiation.

In addition, we observed a similar trend among genes involved in lignin biosynthesis (Fig. 2d and Table S5). Previous studies have reported that disrupted abscission can result from either excessive lignification within the AZ (Li et al. 2006; Lee 2019) or from inadequate lignin barrier formation, which is crucial for restricting the diffusion of cell wall-degrading enzymes during abscission (Lee et al. 2018). Taken together, these findings suggest that altered expression of lignin-related genes may be a potential cause of the impaired seed abscission observed in SW195.

### Altered pectin status underlies delayed abscission in SW195

Our transcriptome analyses indicated that differential regulation of pectin-related genes may contribute to the strong inhibition of seed abscission in SW195. We first compared pectin status between IT030100 and SW195 using ruthenium red staining, which preferentially stains low-methyl-esterified pectins (de Oliveira et al. 2014). SW195 showed stronger staining in both the placenta and seed-side AZ than IT030100 (Fig. 3, a–c), suggesting that SW195 contains a higher level of low-methyl-esterified pectins in these tissues.

**Figure 3:**
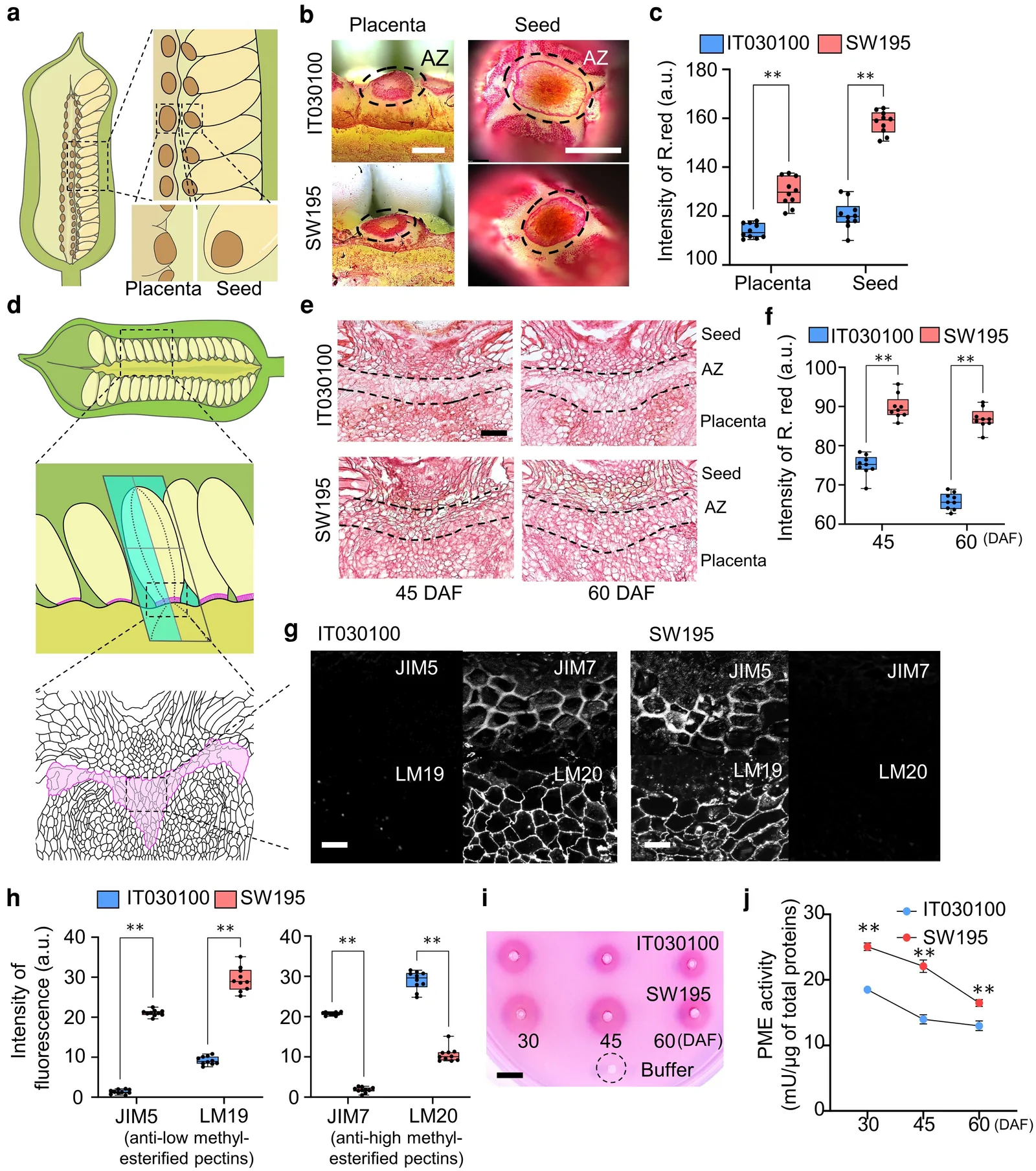
Changes in pectin composition driving AZ-specific cell wall remodeling in sesame. a) Schematic diagram of the seed AZ of sesame depicted in (b, c). b) Visualization of the low-methyl-esterified pectin cell wall in the AZ of the placenta (left) and on the seed-side (right) as visualized by ruthenium red staining at 60 DAF. c) Quantification of ruthenium red staining shown in (b) (indicated black circles). *n* = 10 capsules. d) Schematic diagram of the seed AZ shown in section in (e–g). e) Representative images of the seed AZ of IT030100 and SW195 at the indicated stage. The low-methyl-esterified pectin cell walls were stained using ruthenium red. f) Quantification of ruthenium red staining intensity of the AZ shown in (e). g) Representative immunoﬂuorescence images of AZs probed with the antibodies JIM5, LM19, JIM7, LM20 in IT030100 and SW195 at 45 DAF. White signal indicates antibody labeling. h) Quantiﬁcation of ﬂuorescence in the AZ shown in (g). *n* = 10 capsules. i) Radial gel diffusion assay showing PME activity in proteins extracted from sesame seeds. Three micrograms of total protein, quantified using Bradford protein assays, were loaded in each well. j) Quantiﬁcation of PME activity shown in (i). *n* = 10 biological replicates. All experiments were independently repeated at least 3 times. Error bars represent mean ± SD. **P < 0.01; Student's *t*-test (c, f, h, j). a.u., arbitrary units. Bars indicate 1 mm (b), 100 µm (e, g), 5 mm (i).

To track pectin-related wall changes during the abscission process, we performed ruthenium red staining on sections at 45 and 60 DAF (Fig. 3, d–f). At 45 DAF, the AZ of IT030100 showed weaker staining intensity than surrounding tissue, and the AZ signal further decreased by 60 DAF (Figs. 3, d–f, and S2a). In contrast, the SW195 did not show an AZ-specific decrease at 45 DAF, and the AZ retained strong staining at 60 DAF (Figs. 3, d–f, and S2a). These staining patterns suggest cultivar-dependent differences in pectin-related wall properties in the AZ.

We next assessed pectin methyl-esterification more directly by immunostaining with homogalacturonan (HG) methyl-esterification antibodies. IT030100 showed stronger signals with JIM7 and LM20 (highly methyl-esterified HG), whereas SW195 showed stronger staining with LM19 and JIM5 (low-methyl-esterified HG) (Fig. 3, g and h). Because a low degree of methyl-esterification does not necessarily indicate pectin degradation, and PG activity is a major determinant of HG disassembly in AZ tissues (Ogawa et al. 2009; Daher and Braybrook 2015), we additionally measured PG-associated pectin turnover enzymatically. In IT030100, reaction products (expressed as D-galacturonic acid equivalents) increased during seed development and peaked at 60 DAF (Fig. S2b), whereas SW195 maintained consistently lower product levels throughout the developmental stages. Together, these data suggest that SW195 retains more low-methyl-esterified pectin in the AZ, while showing lower PG-associated pectin turnover compared with IT030100, which may contribute to delayed abscission.

Notably, pectin demethyl-esterification can lead to two distinct outcomes with opposing effects on wall properties: in some contexts, it facilitates HG cleavage by PGs and pectin lyases, whereas in others it promotes Ca^2+^-mediated cross-linking of demethyl-esterified HG, increasing wall rigidity (Wormit and Usadel 2018). In this context, the combination observed in SW195—higher low-methyl-esterified HG signals with lower PG-associated turnover—is consistent with a remodeling state that is not efficiently coupled to HG hydrolysis and may instead favor wall stabilization.

To test whether elevated PME activity could underlie the increased low-methyl-esterified pectin in SW195 (Micheli 2001; Mohnen 2008; Guo et al. 2022), we measured PME activity in vitro. Consistent with the staining patterns, SW195 showed higher PME activity than IT030100 (Figs. 3, i and j, and S2c). We further inhibited PME activity using epigallocatechin gallate (EGCG). EGCG treatment did not measurably change the pectin staining pattern in SW195, whereas it markedly reduced staining intensity in IT030100 (Fig. S2, d–i). These results suggest that inhibiting PME activity alone is not sufficient to shift the pectin-related wall state in SW195, and that additional regulatory components likely contribute to the altered pectin remodeling associated with delayed abscission in this cultivar.

### Lignin–pectin interactions coordinate seed abscission

Our RNA-seq analysis suggested that lignin also contribute to seed abscission in SW195. To examine whether SW195 and IT030100 showed actual differences in lignin accumulation patterns, we performed phloroglucinol-HCl staining on their AZs. In IT030100, staining was strongly detected in the seed-side AZ, whereas in SW195, staining of this area was notably weaker (Fig. 4, a and b). Lignin was hardly detected in either cultivar before 45 DAF (Figs. 4c and S3, a–c), while by 60 DAF, when active abscission begins, lignin began to accumulate near the AZ in IT030100, but not in SW195 (Figs. 4c and S3, a–c).

**Figure 4:**
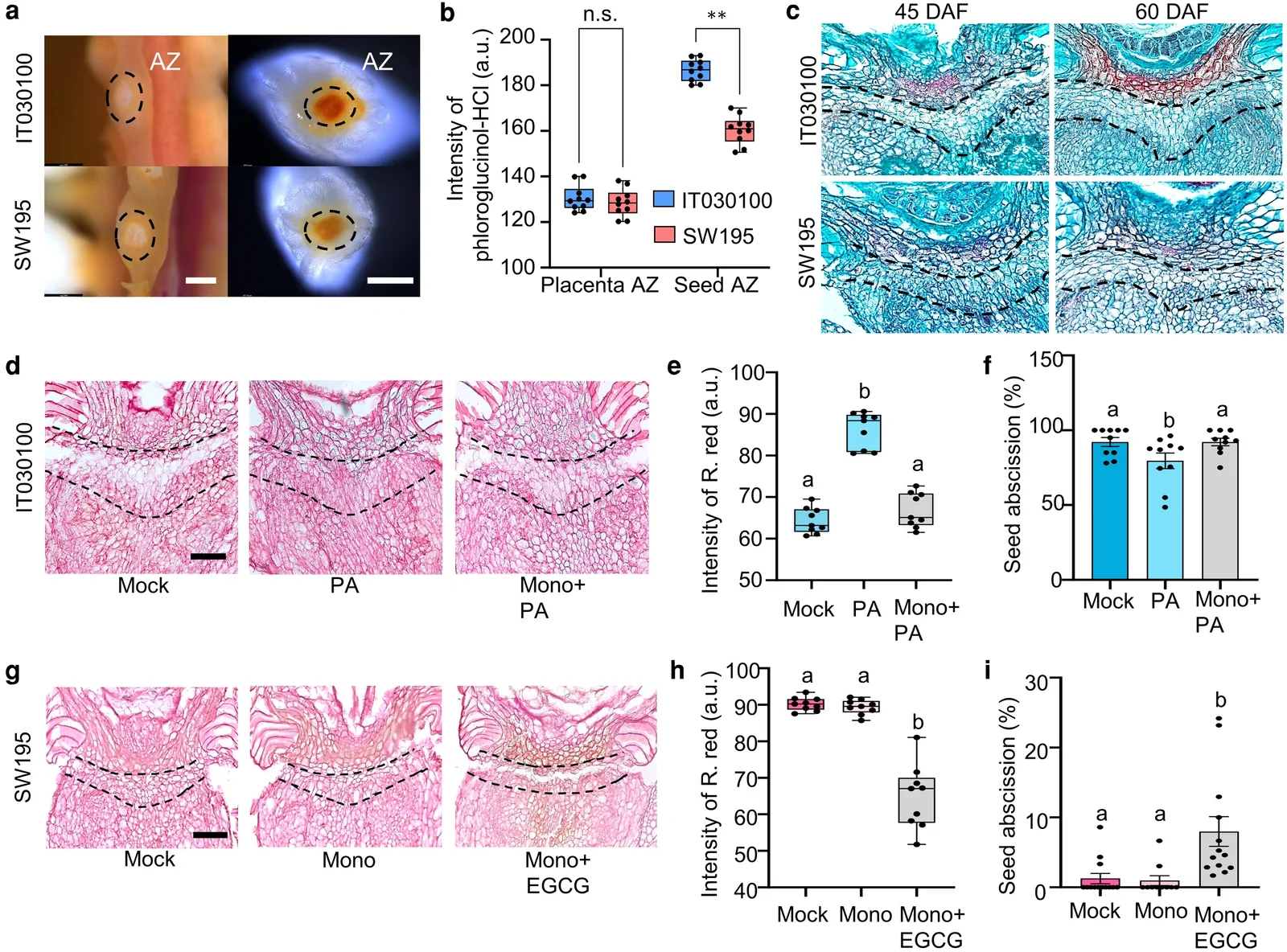
Seed-side AZ-specific lignin composition is required for seed abscission. a) Visualization of lignin cell wall in the AZs of the placenta (left) and on the seed-side (right) using phloroglucinol-HCl at 60 DAF. b) Quantification of phloroglucinol-HCl staining shown in (a, indicated by black circles). *n* = 10 capsules. c) AZ phenotypes in IT030100 and SW195 at the indicated stage. Lignin cell walls were visualized by safranin. d–i) Analysis of seed abscission following treatment with the lignin biosynthesis inhibitor PA (d–f) in IT030100, EGCG (g–i) in SW195 and with or without 100 µM monolignols at 60 DAF. Drugs were mixed with lanolin wax and then placed on the petioles of 30 DAF capsules. Thirty days after treatment, low-methyl-esterified pectin cell walls were visualized by ruthenium red (R. red) (d, g) and quantiﬁed by measuring the intensity of R. red in the AZ (e, h). *n* = 10 capsules (e, h). f, i) Quantification of seed abscission phenotypes shown in (d, g) at 100 DAF. *n* = 10 capsules (f, i). All experiments were independently repeated at least 3 times. Error bars represent mean ± SD. **P < 0.01; Student's *t*-test (b). Different letters indicate signiﬁcant differences among group means. *P* < 0.05, one-way ANOVA with post hoc Tukey HSD test (e) and Kruskal–Wallis with Dunn's multiple-comparison test (f, h, i). n.s., not significant. a.u., arbitrary units. Bars indicate 1 mm (a) or 100 µm (c, d, g).

This staining pattern disappeared following treatment with piperonylic acid (PA), a lignin biosynthetic inhibitor, and was restored by the application of lignin precursors (eg monolignol), thus confirming that the detected signal corresponded to lignin (Fig. S3, d and e). Monolignol treatment also partially restored lignification in SW195 (Fig. S3f), which suggested that the lignin deficiency observed in SW195 is primarily due to impaired monolignol synthesis and not due to defects in lignin polymerization. Taken together, these results suggest a potential role for lignin in sesame seed abscission and imply that altered lignin deposition may contribute to the placental adhesion phenotype observed in SW195.

Next, we considered how lignification in the AZ influences the abscission process. Previous research on floral organ abscission in *Arabidopsis thaliana* suggested that lignin acts as a spatial barrier, where it acts to limit the diffusion of cell wall-degrading enzymes and confines their activity within the AZ (Lee et al. 2018). To determine whether lignin serves a similar role in sesame, we examined the placental surface using scanning electron microscopy (SEM). In IT030100, the separated surface was smooth, whereas in SW195, it was rough and uneven (Fig. S3g). This contrast supports the idea that, as in Arabidopsis, in sesame the accumulation of lignin in the AZ facilitates proper cell layer separation during seed abscission.

Since PME dysregulation is a major issue for SW195, we hypothesized that lignin-mediated compartmentalization may influence PME and/or PMEI activity, thereby regulating pectin modification during abscission. To test this hypothesis, we analyzed pectin staining patterns under conditions that modulate lignin levels. In IT030100, PA treatment significantly increased pectin staining intensity in the AZ, while cotreatment with monolignol restored staining intensity to the level of the untreated control (Fig. 4, d and e). Similarly, PA-treated capsules exhibited reduced seed abscission, whereas cotreatment with monolignol rescued abscission rate (Fig. 4f). Overall, these results suggest that lignin formation in IT030100 is functionally linked to PME activity and abscission progression.

Finally, we note that in SW195, monolignol treatment by itself did not induce noticeable differences in pectin staining (Fig. 4, g and h). However, a combined treatment comprising EGCG and monolignol significantly reduced pectin staining intensity (Fig. 4, g and h) and increased seed abscission rate (Fig. 4i). These results suggest that seed-side lignin formation plays a critical role in regulating PME activity and that the impaired seed abscission in SW195 may result from desynchronized lignin deposition and PME activity.

### QTL mapping and candidate gene identification for seed abscission in SW195

To identify the genetic basis of impaired seed abscission in SW195, we generated 237 recombinant inbred lines (RILs) from a cross between SW195 and IT030100. Seed shattering was evaluated using a 1 to 9 scoring scale, in which score 1 represents the SW195-like nonabscission phenotype and score 9 represents the IT030100-like complete-abscission phenotype (Fig. 5a). The RIL population showed a bimodal distribution, with most lines clustering at the parental extremes and relatively few lines showing intermediate scores, indicating that the trait was expressed predominantly as discrete phenotypic classes. Whole-genome resequencing was performed on both parents and a subset of 100 RILs, generating 20 Gb (78× coverage) for SW195, 18 Gb (73× coverage) for IT030100, and an average depth of 16× for the RILs, based on the published genome size of sesame (Table S6). After mapping to the reference genome, we identified 363,739 variants across 13 chromosomes (316,999 SNPs and 46,740 indels; Table S7). From parental polymorphisms, we selected 3,616 high-confidence SNP markers. After binning to reduce redundancy, 1,859 bin markers were retained and used to construct a genetic linkage map based on recombination breakpoints among the RILs. This final map spanned 1,900 cM with an average marker interval of 1.1 cM (Fig. S4a and Table S8).

**Figure 5:**
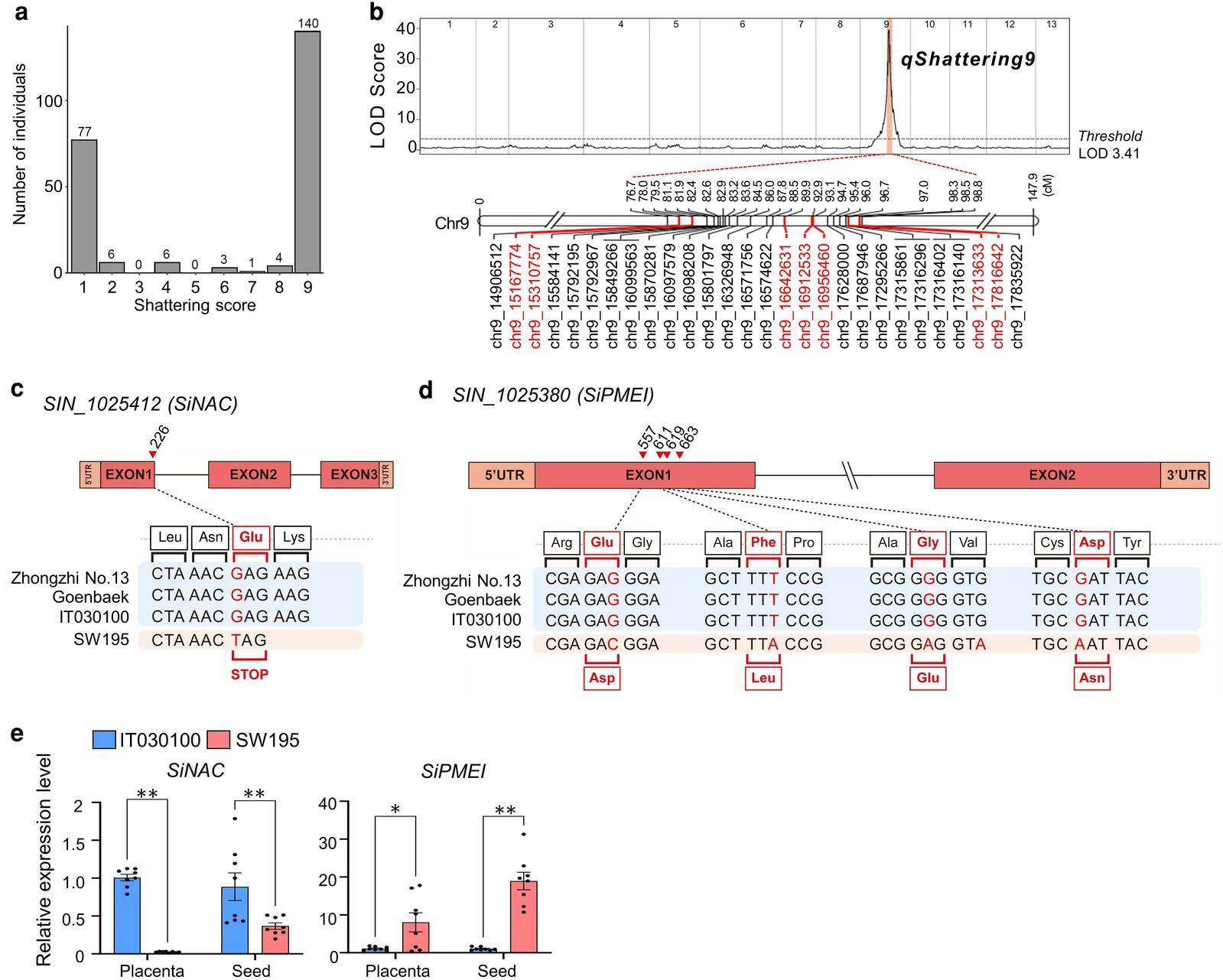
QTL analysis of nonseed abscission traits detected in sesame. a) Distribution of seed abscission scores among 237 RILs derived from a cross between SW195 and IT030100. Seed abscission was evaluated using a manual-shaking assay, as described in the Methods, using a 1 to 9 scale, where score 1 indicates the SW195-like nonabscission phenotype and score 9 indicates the IT030100-like complete-abscission phenotype. Scores 2 to 8 represent intermediate seed release. b) QTL mapping of seed abscission scores using a subset of 100 RILs selected for extreme phenotypes (scores 1 or 9) and IM. The horizontal dashed line indicates the genome-wide significance threshold (LOD = 3.41) determined by 1,000 permutation tests. Red labels indicate markers flanking the significant QTL region on chromosome 9. c, d) Structural diagrams of candidate genes *SiNAC* (c) and *SiPMEI* (d), also shown are differences between shattering cultivars (IT030100 and Goenbaek) and SW195. See Table S12. e) Relative expression levels of candidate genes in IT030100 and SW195 plants as determined by RT-qPCR. 18S ribosomal RNA (rRNA) was used as a reference gene. Values represent the mean ± standard error of the mean (SEM) of three independent experiments. *n* = 3 biological replicates. *P < 0.05, **P < 0.01, Student's *t*-test (e).

We next performed QTL mapping using the shattering scores and the genetic map. A single major QTL peak associated with seed shattering was detected on chromosome 9, with the peak located near chr9_16912533 (Fig. 5b). This QTL exceeded the genome-wide significance threshold (permutation test; see Materials and Methods) and explained 14.2% to 22.2% of the phenotypic variance (Table S9). The additive effect was consistently negative, indicating that the SW195 allele reduces seed abscission. To prioritize candidate variants within this region, we complemented QTL mapping with an allele-frequency contrast between RILs showing strong abscission versus impaired abscission and examined segregation patterns across the chromosome 9 interval (Figs. 5b and S4b). Using an allele-frequency threshold of ≥0.9, we identified 1,027 SNPs within chr9_15575272–chr9_16402088 (Table S10), which we designated as *qShattering9*.

We next annotated sequence variation within *qShattering9* to identify candidate genes for placental adhesion. Excluding intergenic regions, a total of 575 SNPs were located in the ±1 kb putative regulatory regions of 44 genes (Fig. S4b), and 286 SNPs were found within coding sequences, including 49 missense and 2 stop-gain mutations (Tables S11 and S12). Based on functional annotation, we prioritized two candidate genes: *SiNAC* encoding a NAC-domain transcription factor (Arabidopsis AT5G64530.1; SIN_1025412), and *SiPMEI* encoding a pectin methylesterase inhibitor (Arabidopsis AT5G09760.1; SIN_1025380). In SW195, *SiNAC* carried a G-to-T substitution at 226 bp in the first exon that introduces a premature stop codon (Fig. 5c). In *SiPMEI*, four nucleotide substitutions (557, 611, 619, and 663 bp) were detected, resulting in amino-acid changes (Glu → Asp, Phe → Leu, Gly → Glu, and Asp → Asn; Fig. 5d).

To further evaluate the relevance of *SiNAC* and *SiPMEI* to the seed abscission phenotype, we performed reverse transcription-quantitative PCR (RT-qPCR) analysis and developed Kompetitive Allele-Specific PCR (KASP) markers targeting the identified SNPs (Fig. S4). RT-qPCR showed that *SiNAC* expression was significantly downregulated in SW195 relative to IT030100, whereas *SiPMEI* expression was upregulated (Fig. 5e). In addition, genotyping using the KASP markers showed ∼90% concordance with phenotypic classification in the RIL population and ∼95% concordance across 80 sesame accessions (Fig. S4, c–f). Collectively, these results identify *qShattering9* as a major locus associated with seed abscission and support *SiNAC* and *SiPMEI* as promising candidate genes underlying the impaired seed abscission phenotype in SW195.

### SiNAC is associated with lignin-related outputs in sesame

The identification of *SiNAC* as a candidate gene associated with impaired seed abscission in SW195 is noteworthy because it provides a potential link to lignin biosynthesis. *SiNAC* is a putative ortholog of *Arabidopsis XND1*, a NAC-domain transcription factor implicated in regulating secondary wall-related processes in xylem cells (Fig. S5, a and b, and Table S13) (Zhao et al. 2008; Zhong et al. 2021). Although SiNAC retains the conserved NAC domain and several C-terminal motifs found in XND1, sequence divergence in its C-terminal region suggests that its regulatory activity may not be identical to that of XND1.

To characterize *SiNAC*, we transiently expressed *GFP-SiNAC* (Yun et al. 2023) in sesame leaf epidermal cells via *Agrobacterium*-mediated infiltration (Figs. 6a and S5c). GFP-SiNAC signals were predominantly nuclear (Fig. 6b), consistent with its predicted function as a transcription factor. We next examined whether *SiNAC* expression is sufficient to induce lignification-related outputs *in planta*. In sesame leaves transiently expressing *SiNAC*, lignin autofluorescence and phloroglucinol staining were both increased relative to *GFP* controls (Figs. 6, c and d, and S5, d and e).

**Figure 6:**
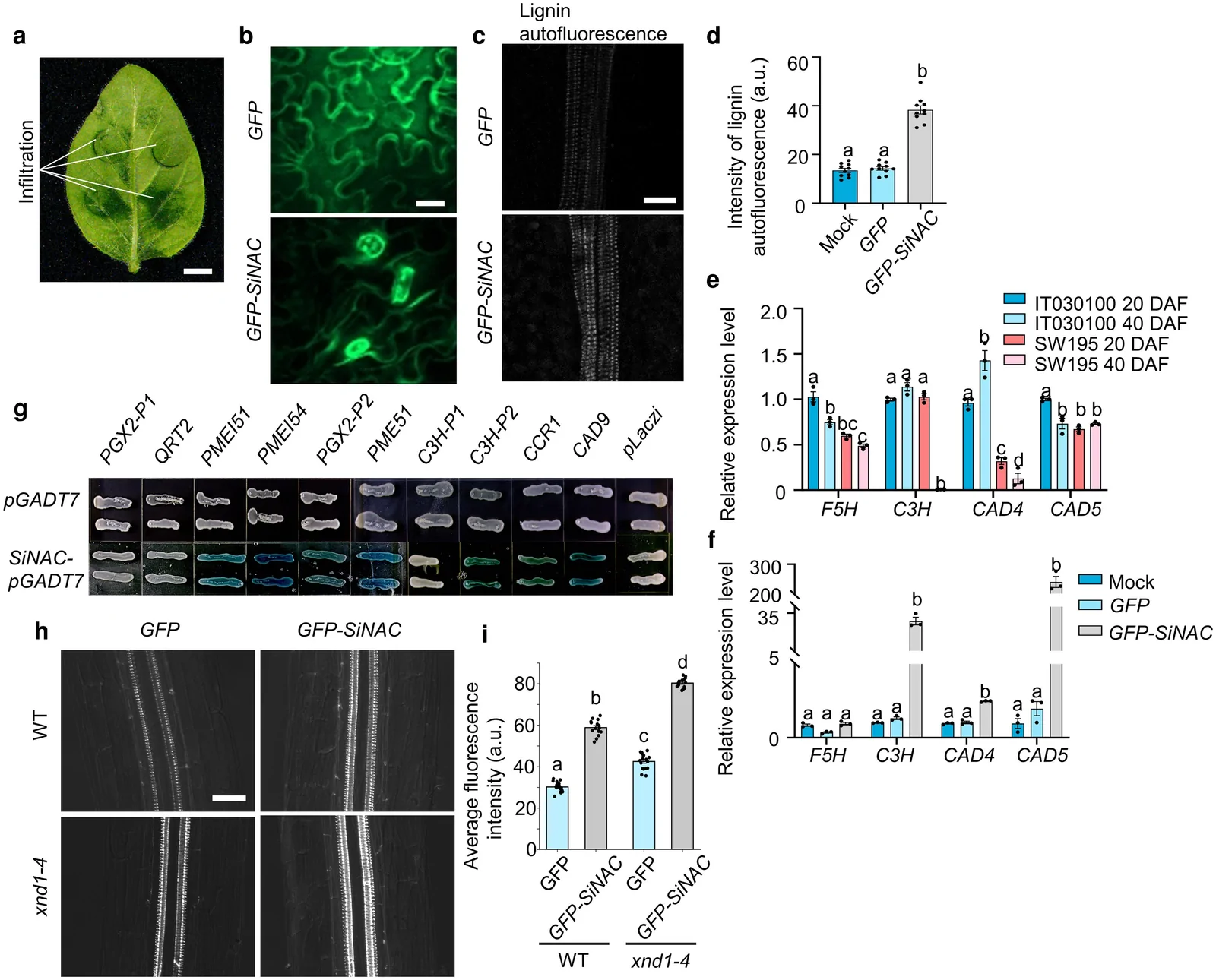
SiNAC modulates lignin formation in sesame. a) Schematic view of sesame leaves transiently infiltrated via Agrobacterium-mediated infiltration. b) Subcellular localization of SiNAC protein in sesame. GFP fluorescence was detected in leaves infiltrated with Agrobacterium carrying *MacT::GFP* overexpression(*GFP* OE) (upper panel) or *MacT::GFP-SiNAC* (*SiNAC* OE, lower panel) plasmids. GFP signals were then observed in the infiltrated region of the leaf indicated in (a). c) Maximum projection images produced by confocal microscopy show lignin autoﬂuorescence in infiltrated leaves following induction by UV. Leaves were cleared prior to autoﬂuorescence analysis. d) Quantification of autoﬂuorescence intensity shown in (c). *n* = 10 leaves. Error bars represent mean ± SD. e, f) Relative expression levels of indicated genes in the seed-side AZ of IT030100 and SW195 at the indicated stages (e) and in the indicated agrobacterium-infiltrated sesame leaves (f), as determined by RT-qPCR. 18S rRNA was used as the reference gene. Values represent the mean ± standard error of the mean (SEM) of three independent experiments. *n* = 3 biological replicates. g) Yeast one-hybrid (Y1H) binding assay of SiNAC to promoter regions associated with pectin and lignin biosynthesis. Yeast strain EGY48 cells were cotransformed with the LacZ reporter gene driven by the indicated promoter fragments cloned into pLacZi (bait) and either empty pGADT7 (AD) or *SiNAC* fused to pGADT7 (*SiNAC*-pGADT7, prey). The empty pGADT7 and pLacZi vectors were used as negative controls. Transformants were grown on SD/-Ura-Leu dropout plates containing X-gal, and protein–DNA interactions were determined based on blue color development. h) Confocal microscopy images of lignin deposition in the roots of Arabidopsis plants overexpressing *SiNAC*. Roots were stained with Basic fuchsin and imaged at 15 DAG. Images were captured at the beginning of the differentiation zone. i) Quantification of basic fuchsin signal intensity in (h). *n* = 15 roots from two independent lines. Error bars represent mean ± SD. All experiments were independently repeated at least 3 times. Different letters indicate signiﬁcant differences among group means. *P* < 0.05, Kruskal–Wallis with Dunn's multiple-comparison test (d, i) and one-way ANOVA with post hoc Tukey HSD test (e, f). a.u., arbitrary units. Bars indicate 2 mm (a), 100 µm (b), 200 µm (c, h).

To determine whether this lignification-promoting activity was accompanied by changes in lignin biosynthetic gene expression, we identified sesame homologs of *Arabidopsis* monolignol biosynthetic genes and examined their expression in seed-side AZ samples (Figs. 6e and S6a, and Table S14). Consistent with the reduced lignification observed in SW195, several lignin biosynthetic genes, including *F5H*, *C3H*, *CAD4*, and *CAD5*, were significantly downregulated in SW195 relative to IT030100 (Fig. 6e). Conversely, these genes were upregulated in *SiNAC*-expressing leaves (Fig. 6f), further supporting a positive role for SiNAC in lignin biosynthetic gene expression.

Notably, *XCP1*, a direct target repressed by *Arabidopsis* XND1, and several other XND1-repressed genes were also downregulated in SW195 relative to IT030100 (Fig. S6b and Table S15) (Zhao et al. 2017; Zhong et al. 2021; Ding et al. 2025). Their downregulation in SW195 parallels that of lignin biosynthetic genes and is therefore consistent with a role for SiNAC in promoting their expression. Thus, despite its close relationship to XND1, SiNAC may differ from XND1 in its regulatory relationship with a subset of XND1-responsive genes. Sequence divergence in the C-terminal region may contribute to this functional distinction, although this possibility remains to be tested experimentally.

To identify candidate cell wall-related genes that may be regulated by SiNAC, we screened the promoters of lignin- and pectin-related genes for predicted NAC-binding motifs (Tran et al. 2004; Chun et al. 2023). Multiple predicted NAC-binding motifs were identified in these promoter regions (Fig. S6c). Promoter fragments containing these motifs were fused to a *LacZ* reporter and tested by yeast one-hybrid assays. Several pectin- and lignin-related promoter fragments showed SiNAC-dependent reporter activation (Fig. 6g). This result was further supported by quantitative chlorophenol red-β-D-galactopyranoside (CPRG) assays, which showed significantly higher β-galactosidase activity for the *PMEI51*, *PMEI54*, *PGX2-P2*, *PME51*, *C3H-P2*, and *CAD9* promoter fragments in the presence of SiNAC than in the empty activation domain (AD) control (Fig. S6d). These results identify SiNAC as a candidate regulator of lignin- and pectin-related gene expression, while its direct regulatory functions in the seed AZ remain to be established.

In a heterologous context, *SiNAC* overexpression increased secondary cell wall-associated lignin deposition in both wild-type *Arabidopsis* and *xnd1-4* background relative to *GFP* controls (Fig. 6, h and i). Thus, SiNAC can promote lignin-associated outputs in both sesame and *Arabidopsis*. Together, these results support SiNAC as a candidate regulator of lignin- and pectic-related transcriptional programs and a potential contributor to cell wall-associated changes in SW195.

## Discussion

Abscission is a fundamental process that plants use to enable the timely removal of senescent or damaged organs and to facilitate efficient seed dispersal (Addicott 1982; Roberts et al. 2002). This process is tightly regulated by a complex interplay of hormonal signaling and transcriptional networks that coordinate the localized degradation of the middle lamella in specific cell layers of the AZ. Although studies of *Arabidopsis* floral organ abscission have identified key signaling pathways and cell wall-modifying enzymes (Yu and Kellogg 2018; Kim et al. 2019b; Lee 2019; Wu et al. 2023b; Li and Su 2024), the mechanisms controlling seed abscission in crops remain less well understood. Here, comparison of the nonshattering sesame line SW195 with the shattering cultivar IT030100 revealed altered pectin-related wall remodeling and reduced seed-side lignification in the AZ of SW195. These features were associated with delayed seed abscission, indicating that multiple cell wall-related processes contribute to the strong placenta adhesion phenotype of SW195.

Spatially restricted lignification appears to be an important, although anatomically variable, feature of abscission. In Arabidopsis, lignin contributes to floral organ abscission by serving as an apoplastic barrier that spatially restricts the diffusion of cell wall-degrading enzymes, thereby enabling precise organ separation (Lee et al. 2018). Seed abscission in *Arabidopsis* also requires spatially patterned lignification at the funiculus-seed junction, as either loss of the lignified layer or ectopic lignification of the separation zone impairs detachment (Balanzà et al. 2016). In rice, by contrast, shattering cultivars form a lignin-free layer at the AZ, whereas nonshattering cultivars retain lignification in this region (Yoon et al. 2017; Wu et al. 2023a). These contrasting examples indicate that the effect of lignin on abscission depends on its location and developmental context. In sesame, lignin accumulated predominantly in the seed-side AZ, and perturbation of lignin biosynthesis altered pectin staining and seed abscission. Thus, although the lignified domains differ anatomically between Arabidopsis and sesame, spatially restricted lignification may influence seed abscission in both species. Our perturbation experiments support a functional association between lignification, pectin-related wall remodeling, and seed abscission in sesame, but the underlying mechanism remains to be determined.

Pectin remodeling represents an additional component of the SW195 phenotype. HG is secreted in a highly methyl-esterified state and is subsequently modified by pectin methylesterases (PMEs) and pectin methylesterase inhibitors (PMEIs) (Mohnen 2008; Su 2023). The balance between PME and PMEI activities can affect cell adhesion, organ separation, and other developmental processes (Mohnen 2008; Qi et al. 2017; Jonsson et al. 2021). SW195 showed higher PME activity and stronger accumulation of low-methyl-esterified HG, together with lower PG-associated pectin turnover, than IT030100. This combination is consistent with a pectin-remodeling state that is not efficiently coupled to HG hydrolysis and may favor wall stabilization in the AZ. However, the causal relationships among PME activity, HG status, and delayed seed abscission will require further investigation.

QTL mapping and whole-genome resequencing identified *SiPMEI* and *SiNAC* within *qShattering9* as candidate genes associated with the SW195 phenotype. The sequence and expression differences observed for *SiPMEI* make it a plausible candidate associated with the altered pectin-related wall state in SW195, although its functional contribution has not yet been established. The broad expression changes observed among pectin- and lignin-related genes additionally raise the possibility that an upstream transcriptional regulator contributes to the phenotype. In this context, *SiNAC* is a plausible candidate because its expression differed between the two cultivars, transient *SiNAC* overexpression enhanced lignin accumulation, and yeast one-hybrid assays showed binding of SiNAC to promoter fragments of lignin- and pectin-related genes. These observations do not establish direct regulation of pectin remodeling or seed abscission by SiNAC, but they support its further evaluation as a candidate regulator of cell wall-related gene expression.

Phylogenetic analysis placed *SiNAC* within the XND1 clade, supporting its assignment as a putative ortholog of *Arabidopsis XND1*, a NAC-domain transcription factor implicated in xylem differentiation and secondary cell wall formation (Zhao et al. 2008; Zhong et al. 2021). However, SiNAC diverges from XND1 in its C-terminal region, which is required for XND1-mediated repression. In *Arabidopsis*, XND1 directly binds to the *XCP1* promoter and represses its activity (Ding et al. 2025), and it also inhibits vascular-related NAC-domain/secondary wall NAC-mediated transcriptional activation of other secondary wall-associated genes, including downstream transcriptional regulators such as *SND2*, *SND3*, and several *MYB* genes (Zhao et al. 2017; Zhong et al. 2021). In contrast, sesame homologs of these XND1-repressed genes were downregulated in SW195, which carries a predicted loss-of-function mutation in *SiNAC*. Together with the increased lignin accumulation observed following transient *SiNAC* overexpression, these findings suggest that SiNAC may have regulatory outputs distinct from those of XND1. The divergent C-terminal region may contribute to this difference, although this possibility requires experimental validation. Identifying the direct targets and protein interaction partners of SiNAC will be important for defining its role in the seed AZ.

In conclusion, our study identifies altered pectin-related wall remodeling and reduced seed-side lignification as features associated with delayed seed abscission in SW195. The identification of *SiNAC* and *SiPMEI* as candidate genes provides a foundation for future functional studies to determine their respective and potentially combined contributions to seed abscission. These findings also offer a framework for developing reduced-shattering sesame cultivars with improved harvestability.

## Materials and methods

### Plant materials and growth conditions

Plants from two sesame (*S. indicum*) cultivars, ie IT030100 (Early Russian) and Suwon195 (SW195), were cultivated in a greenhouse under day and night temperatures of 30 °C and 20 °C and a relative humidity of 50%. We used 2 parent lines of sesame, IT030100 and SW195, for crossing to generate RILs. A total of 237 plants in the F8 generation were developed from a single seed with selfing during subsequent generations. Phenotyping for nonabscission was conducted in 2018 using a randomized complete block design that included 3 technical replicates each containing 237 RILs. Seed shattering was evaluated after gentle hand agitation using an ordinal 1 to 9 scale. SW195, which retains its seeds, was assigned the lowest value (1), while IT030100, characterized by complete seed release, was assigned the highest value (9). Dried pods were placed in Falcon tubes and subjected to mechanical impact, after which seed release was assessed. SW195, the nonabscission type, released approximately 5% to 10% of its seeds and was assigned a score of 1, whereas IT030100 released more than 50% and was assigned a score of 9. Genotypes with intermediate levels of seed release were assigned relative scores between 1 and 9.

For Arabidopsis, 15 days after germination (DAG) *Arabidopsis thaliana* (accession Col-0) plants were used. After stratification for 2 days (at 4 °C in the dark), seeds were sown on a soil:sand mixture (4:1, w/w) and cultivated in a climate chamber with 60% relative humidity under long-day conditions (16-h-light/8-h-dark cycles, 22 °C day/18 °C night regime, 70 μmol m^−2^ s^−1^ photon flux density).

### Plasmid construction and plant transformation

For transient expression assays, full-length *SiNAC* complementary DNA (cDNA) sequences were first cloned into pCAMBIA-1300 vectors containing GFP, and expression of cloned genes was driven by the MacT promoter and RD29b terminator (Yun et al. 2023). The resulting binary vectors were then transformed into *Agrobacterium tumefaciens* (Agrobacterium) strain EHA105 for transient gene expression in sesame plants. Transformation strains of Agrobacterium were grown at 28 °C in LB medium supplemented with rifampicin and kanamycin (both at 50 µg/ml). Cultures were then diluted to an OD600 of 0.2 in an infiltration solution containing 10 mM MES (pH 5.6), 10 mM MgSO_4_ and 400 µM acetosyringone, before being infiltrated in the leaves of growth chamber-grown sesame plants using a syringe (without a needle). Specifically, the first and second main leaves of 10 DAG plants were infiltrated with *MacT::GFP* and *MacT::GFP-SiNAC*, respectively. Arabidopsis transgenic lines were generated by the floral-dip method using Agrobacterium tumefaciens strain GV3101. For each construct, two independent transgenic lines were analyzed. The phenotypic and histological analyses were performed using homozygous T2 plants from two independent lines.

### Immunoﬂuorescence

Cell wall immunoﬂuorescence was quantified following a previously described protocol (Pasternak et al. 2015; Shimamura 2015). Briefly, to calculate the average wall intensity of each probe, the probe signal within the AZ was divided by the area of cell wall autoﬂuorescence using FIJI version 2.9.0. We used 10 biological samples per genotype and measured the AZ for all samples. Speciﬁcally, AZs were dissected at 45 DAF then ﬁxed in freshly prepared 4% (w/v) paraformaldehyde (PFA) in 1× phosphate-buffered saline (PBS) for 12 h. Samples were then dehydrated using an ethanol series, embedded in parafﬁn, then sectioned. To determine immunoﬂuorescence, samples were deparafﬁnized, rehydrated, and incubated in 10% (w/v) bovine serum albumin (BSA) in TBS buffer for 1 h at RT. Monoclonal HG antibody (kerafast, ELD001, 003, 004, 005) was diluted by a ratio of 1:50 in 10% (v/v) BSA and TBS. The working antibody solution was added to samples, which were left to incubate for 1 h at RT. Samples were then washed three times for 5 min, then incubated in Alexa Fluor® 568 goat antimouse antibody (1:500 dilution) (Thermo Fisher Scientiﬁc, A-11004) for 1 h at RT. Finally, samples were rinsed and mounted in TBS, then imaged for ﬂuorescence. Alexa Fluor® 568 was detected at an excitation wavelength of 579 nm and an emission wavelength window of 580–620 nm.

### Histological staining and microscopy

For ruthenium red staining, capsules were immersed in a 0.02% (w/v) ruthenium red solution prepared in water for 20 min, then rinsed for 5 min in distilled water. For phloroglucinol-HCl staining, capsules were first incubated in 70% ethanol for 5 days, then stained with a 3% phloroglucinol solution in 100% ethanol for another 3 days. Immediately before imaging, samples were treated with 37% HCl. Low-magnification images were then collected using a Leica stereomicroscope (M205FA, Leica) and confocal laser-scanning microscopy images were obtained using a Zeiss LSM 700 (Zen SP3 Black edition). The excitation and emission windows were as follows: GFP: 488 nm and 500 to 530 nm, Basic Fuchsin: 561 nm and 600 to 630 nm.

For SEM, we first placed seeds from specified plants under a vacuum for 30 min in a 4% PFA solution. After washing with PBS, their cytoplasmic content was extracted using 1% Triton X-100 (Sigma, T8787) for 72 h. Samples were then postfixed in 1% osmium tetroxide. Following fixation, dehydration was carried out using a graded ethanol series, after which samples were dried using a critical point dryer (Leica). Just before imaging, samples were coated with a thin layer of platinum then observed using a field-emission scanning electron microscope (Zeiss).

### Quantification of pectin methylesterase (PME) activity

PME activity was assessed using a gel diffusion technique adapted from previously published protocols (Downie et al. 1998; Lionetti 2015). Briefly, we first extracted proteins by homogenizing sesame seed tissue in 1 ml of extraction buffer (0.1 M citrate buffer and 0.2 M sodium phosphate [dibasic] at pH 7.0, supplemented with 2.5 mM phenylmethylsulfonyl fluoride). The resulting homogenate was centrifuged at 16,000 × g for 15 min at 4 °C, after which the supernatant was collected. Extract total protein concentration was then measured by Bradford assay using a BSA standard. Next, we loaded aliquots of protein extract (3 µg/10 µl) into 1 mm diameter wells punched into a 2% (w/v) agarose gel containing 0.1% (w/v) apple pectin (Sigma, P8471) cast in a Petri dish. After loading, these gels were incubated at 28 °C for 16 h to allow enzymatic diffusion and pectin de-esterification. Following incubation, all gels were rinsed with distilled water then stained for 30 min at room temperature using 0.05% (w/v) ruthenium red (Sigma, R2751). PME activity was quantified by comparing the diameters of stained halos to a calibration curve generated using a commercial PME standard derived from *Aspergillus aculeatus* (Sigma, P2611).

### Pharmacological assays

To evaluate the impact of chemical treatment on seed abscission, various drugs were incorporated into lanolin wax (Sigma, L7387) that was liquefied at 50 °C. The resulting mixtures were applied to the petioles of seed capsules at 30 DAF using sterile wooden toothpicks. All chemicals were initially prepared as 100 mM stock solutions in dimethyl sulfoxide (Sigma, D8418) then diluted to the following working concentrations: 250 to 500 µM for EGCG (Sigma, E4143), 50 mM for ethylene glycol tetraacetic acid, 500 to 1,000 µM for PA (Sigma, P49805) and monolignols (coniferyl and sinapyl alcohols in PBS at 100 µM each), resulting solutions were applied for 30 days.

### Yeast one-hybrid assay

Yeast one-hybrid assays were performed using a modified system based on previously described methods (Lopato et al. 2006; Kang et al. 2024). Promoter fragments associated with pectin and lignin biosynthesis were amplified and cloned into the pLacZi vector to generate LacZ reporter constructs. The lengths of the promoters used in this experiment and the primer information are provided in Table S16. The reporter constructs were linearized and integrated into the genome of the yeast strain EGY48. The full-length coding sequence of SiNAC was cloned into the pGADT7 vector to generate the activation domain fusion construct (SiNAC-pGADT7). Transformants were selected on SD/-Ura-Leu dropout medium and subsequently transferred to plates containing X-gal (5-bromo-4-chloro-3-indolyl-β-D-galactopyranoside, Sigma, B4252) for detection of reporter gene activation. Protein–DNA interactions were determined based on the development of blue coloration after 3 to 5 days of incubation. For CPRG assay, the bait and prey plasmids were cotransformed into the yeast strain YM4271. β-Galactosidase (β-Gal) activity was quantified using CPRG (Roche, 10884308001), and absorbance was measured at 574 nm using a microplate spectrophotometer (PowerWave X, BioTek). β-Gal activity was normalized to cell density (OD600). For each pLacZi bait construct, β-Gal activity was expressed relative to that of the corresponding bait construct cotransformed with the empty pGADT7 vector, which was set to 1.

### PG activity assay

PG activity was measured using the 3,5-dinitrosalicylic acid (DNSA) method based on the quantification of released D-galacturonic acid. Whole seeds from IT030100 and SW195 were harvested at 30, 45, and 60 DAF and used for protein extraction. The reaction mixture contained polygalacturonic acid (0.5%, w/v) in 100 mM sodium acetate buffer (pH 4.6) and protein extracts in a total volume of 225 μl. The reactions were incubated at 30 °C for up to 6 h. To terminate the reaction, 750 μl of DNSA reagent (Sigma, D0550) was added, followed by incubation at 100 °C for 5 min. After cooling, absorbance was measured at 575 nm using a microplate reader. A standard curve was generated using D-galacturonic acid (Sigma, 48280), and PG activity was calculated based on the amount of reducing sugars released. Each experiment was performed with three independent biological replicates.

### Resequencing and SNP calling

The 2 parental lines and 100 RILs were subjected to whole-genome resequencing. First, genomic DNA was extracted from young leaves using the NucleoSpin Plant II Mini Kit (Macherey-Nagel, Germany), then genomic DNA libraries were prepared using TruSeq Nano DNA High Throughput Library Prep Kits (Illumina, USA) before samples were sequenced on an Illumina HiSeq 2000 platform with 150 bp paired-end reads. Low-quality bases (Phred scores <30) were trimmed using Trimmomatic version 0.33 and the resulting clean reads were aligned to the sesame reference genome (Zhongzhi No. 13, version 2.0) using BWA version 0.7.17. Variant calling was performed with SAMtools version 1.3 and VCFtools, applying the following filters: mapping quality ≥30, read depth ≥3, heterozygosity ≤10%, and missing genotype rate ≤70%. Variants within 100 bp of each other were excluded. Finally, SNP annotation was conducted using SnpEff.

### Linkage mapping and QTL analysis

To address analytical limitations caused by missing genotypes and redundancy due to limited segregation caused by linkage, we performed genotype imputation and binning using Genotype-Corrector (Miao et al. 2018). This process allowed us to select representative, informative markers; once obtained, these markers were used to construct a genetic map using 13 linkage groups. Marker distances were calculated as recombination frequencies (cM) by the Kosambi mapping function implemented in JoinMap version 4.1. Finally, QTL analysis was performed by the interval mapping by Interval mapping for additive QTL method as implemented in IciMapping version 4.4; for this analysis, the log of the odds (LOD) threshold was set after 1,000 permutation tests.

### Candidate gene selection and KASP marker analysis

To identify potential candidate genes associated with the nonabscission trait, we first retrieved a list of genes located within the QTL regions on chromosome 9 from the Zhongzhi No. 13 reference genome (version 2.0). The putative functions of the sesame genes were inferred by identifying orthologs using BLASTp similarity searches against *Arabidopsis thaliana* protein sequences (TAIR version 10). Subsequently, SNPs detected within these genes were functionally categorized using SnpEff software based on their predicted impact on gene structure. These impacts were classified as HIGH, MODERATE, LOW, or MODIFIER. Allele-specific KASP markers targeting SNPs in two candidate genes, *SiNAC* and *SiPMEI* (Table S16), were designed by Biosearch Technologies (California, USA). Genotyping was then conducted using a QuantStudio 5 Real-Time PCR system (Thermo Fisher Scientific, USA), and allele discrimination was analyzed using QuantStudio Design & Analysis. Genotype clustering was performed as per the manufacturer's instructions and was manually curated to ensure accuracy.

### RNA extraction and reverse-transcription RT-qPCR

Seed samples were obtained by manually excising AZ regions from approximately 20 seeds of both IT030100 and SW195 plants. Samples were selected carefully to ensure that the appropriate developmental stage was selected from the primary inflorescence. Next, total RNA was isolated from AZ tissue samples using the RNeasy Plant Mini Kit (Qiagen), with all procedures following the manufacturer's protocol. RNA concentration and purity were assessed using an ND-2000 spectrophotometer (Thermo Fisher Scientific). Extracted RNA showed OD 260/280 ratios ranging between 1.9 and 2.1, with optical density (OD) 260/230 ratios between 2.0 and 2.5. RNA integrity was further evaluated using a TapeStation RNA screentape system (Agilent), and only samples with an RNA integrity numbers (RINs) greater than 9.0 were selected for RNA-seq library preparation.

Next, cDNA synthesis was carried out using 2 μg of total RNA with a first-strand synthesis kit (GenDepot) and oligo(dT) primers, with all procedures following the manufacturer's instructions. cDNA was then used for qPCR analysis performed on a Quant Studio 1 platform (Applied Biosystems) using SYBR Green Real-time PCR Master Mix (Applied Biosystems) (Bong et al. 2024). All primer sequences used for amplification are listed in Table S16. After qPCR, threshold cycle (Ct) values were used to calculate 2^−ΔΔCt^ values to quantify expression levels. ΔΔCt values for treated plants were determined as follows: (Ct target gene—Ct 18S rRNA gene)—control plant (Ct target gene—Ct 18S rRNA gene) (Livak and Schmittgen 2001).

### Transcriptome deep sequencing (RNA-seq) library preparation and sequencing

We also independently prepared RNA-seq libraries using TruSeq Stranded mRNA Library Prep Kits (Illumina) as per the manufacturer's protocol. Library quality and concentration were then evaluated using the KAPA Library Quantification Kit (KAPA BIOSYSTEMS) optimized for Illumina sequencing platforms, with all procedures following the qPCR quantification protocol guide. Only RNA samples with RINs greater than 9.0 were included in subsequent analyses. Sequencing was performed on an Illumina NovaSeq platform (Illumina), generating 150-base pair paired-end reads. Library preparation and sequencing were outsourced to Macrogen (South Korea).

### RNA-seq analysis and functional annotation

After sequencing, TruSeq universal and indexed adapters were removed using Trimmomatic version 0.39 (Bolger et al. 2014). Quality filtering of sequencing reads was performed with PRINSEQ version 0.20.4 (Schmieder and Edwards 2011), and high-quality reads thus obtained were subsequently aligned to the reference genome (Zhongzhi No. 13, version 2.0) using HISAT2 version 2.2.1 (Kim et al. 2019a). Gene abundance was quantified using StringTie version 1.3.4 (Pertea et al. 2015). For this step, we used the TAIR10 genome GFF3 annotation file for reference-based gene identification. The resulting gene expression data were compiled into a matrix representing transcript abundance in transcripts per million. To account for compositional biases across samples, normalization was conducted using the trimmed mean of *M*-values (TMM) method implemented in the edgeR package version 3.19 in R (Robinson et al. 2010). DEGs were identified as having a log_2_ fold-change (log2FC) greater than 0.58 and a *P*-value <0.05. *Z*-score-transformed expression values of the target gene set were visualized as a heatmap using pheatmap package version 1.0.12 in R (Kolde 2019). Subsequently, we subjected all gene lists to GO enrichment analysis via the DAVID platform (Sherman et al. 2022). Finally, Web Gene Ontology Annotation Plot (WEGO) analysis was conducted using WEGO version 2.0 (Ye et al. 2018).

### Statistical analyses

All statistical analyses were performed using Statistical Package for the Social Sciences (SPSS) (version 26, IBM SPSS Inc.). For comparisons between two independent groups, a two-tailed Student's *t*-test was used. To determine the statistical significance of differences in group means, we used one-way analysis of variance (ANOVA) followed by Tukey's honestly significant difference (HSD) test that was used for normally distributed data with homogeneous variances. When these assumptions were not met, the Kruskal–Wallis test followed by Dunn's multiple-comparison test was used. A *P*-value of <0.05 was considered statistically significant. Each experiment was independently repeated three times.

## Supplementary Material

kiag762_Supplementary_Data

## Data Availability

The raw data of DNA sequencing are available on the Sequence Read Archive in the National Center for Biotechnology Information (NCBI) (https://submit.ncbi.nlm.nih.gov/subs/sra/SUB12938631). The RNA-Seq raw data have been deposited at the National Center for Biotechnology Information Sequence Read Archive under accession number PRJNA1219684.

## References

[kiag762-B1] Addicott FT . 1982. Abscission. Univ of California Press.

[kiag762-B2] Balanzà V, Roig-Villanova I, Di Marzo M, Masiero S, Colombo L. 2016. Seed abscission and fruit dehiscence required for seed dispersal rely on similar genetic networks. Development. 143:3372–3381. 10.1242/dev.135202. 27510967

[kiag762-B3] Bolger AM, Lohse M, Usadel B. 2014. Trimmomatic: a flexible trimmer for Illumina sequence data. Bioinformatics. 30:2114–2120. 10.1093/bioinformatics/btu170. PMC4103590 24695404

[kiag762-B4] Bong D, Sohn J, Lee S-JV. 2024. Brief guide to RT-qPCR. Mol Cells. 47:100141. 10.1016/j.mocell.2024.100141. PMC11612376 39476972

[kiag762-B5] Chun I, Kim H, Hong S, Kim Y, Kim M. 2023. Structural basis of DNA binding by the NAC transcription factor ORE1, a master regulator of plant senescence. Plant Commun. 4:100510. 10.1016/j.xplc.2022.100510. PMC10203392 36564947

[kiag762-B6] Daher FB, Braybrook SA. 2015. How to let go: pectin and plant cell adhesion. Front Plant Sci. 6:523. 10.3389/fpls.2015.00523. PMC4500915 26236321

[kiag762-B7] de Oliveira DC et al 2014. Variation in the degree of pectin methylesterification during the development of *Baccharis dracunculifolia* kidney-shaped gall. PLoS One. 9:e94588. 10.1371/journal.pone.0094588. PMC3991646 24747777

[kiag762-B8] Ding B et al 2025. The transcription factors DOF4. 6 and XND1 jointly regulate root hydraulics and drought responses in Arabidopsis. Plant Cell. 37:koaf083. 10.1093/plcell/koaf083. PMC12013818 40262770

[kiag762-B9] Downie B et al 1998. A gel diffusion assay for quantification of pectin methylesterase activity. Anal Biochem. 264:149–157. 10.1006/abio.1998.2847. 9866676

[kiag762-B10] Emebiri L, Hildebrand S. 2023. Natural variation and genetic loci underlying resistance to grain shattering in standing crop of modern wheat. Mol Genet Genomics. 298:1211–1224. 10.1007/s00438-023-02051-z. PMC10363068 37410105

[kiag762-B11] Greenberg J, Goren R, Riov J. 1975. The role of cellulase and polygalacturonase in abscission of young and mature Shamouti orange fruits. Physiol Plant. 34:1–7. 10.1111/j.1399-3054.1975.tb01845.x.

[kiag762-B12] Guo S, Wang M, Song X, Zhou G, Kong Y. 2022. The evolving views of the simplest pectic polysaccharides: homogalacturonan. Plant Cell Rep. 41:2111–2123. 10.1007/s00299-022-02909-3. 35986766

[kiag762-B13] Huberman M, Goren R. 1979. Exo-and endo-cellular cellulase and polygalacturonase in abscission zones of developing orange fruits. Physiol Plant. 45:189–196. 10.1111/j.1399-3054.1979.tb01685.x.

[kiag762-B14] Ishikawa R et al 2022. A stepwise route to domesticate rice by controlling seed shattering and panicle shape. Proc Natl Acad Sci U S A. 119:e2121692119. 10.1073/pnas.2121692119. PMC9245615 35733263

[kiag762-B15] Jiang L et al 2019. The APETALA2-like transcription factor SUPERNUMERARY BRACT controls rice seed shattering and seed size. Plant Cell. 31:17–36. 10.1105/tpc.18.00304. PMC6391694 30626621

[kiag762-B16] Jonsson K et al 2021. Mechanochemical feedback mediates tissue bending required for seedling emergence. Curr Biol. 31:1154–1164.e3. 10.1016/j.cub.2020.12.016. 33417884

[kiag762-B17] Kang J et al 2024. Rice ONAC005-OsMADS50-OsMADS56 interaction downregulates OsLFL1 in promoting long-day-dependent flowering. Crop J. 12:1607–1618. 10.1016/j.cj.2024.10.003.

[kiag762-B18] Kim D, Paggi JM, Park C, Bennett C, Salzberg SL. 2019a. Graph-based genome alignment and genotyping with HISAT2 and HISAT-genotype. Nat Biotechnol. 37:907–915. 10.1038/s41587-019-0201-4. PMC7605509 31375807

[kiag762-B19] Kim DH, Chae YA, Kang CW, Park CH, Seong NS. 2004. Grain shattering resistance and its screening method of sesame. Korean J Crop Sci. 49:491–495. https://koreascience.kr/article/JAKO200431234707061.page

[kiag762-B20] Kim J, Chun J-P, Tucker ML. 2019b. Transcriptional regulation of abscission zones. Plants. 8:154. 10.3390/plants8060154. PMC6631628 31174352

[kiag762-B21] Kolde R . 2019. pheatmap: Pretty Heatmaps. R package version 1.0. 12.

[kiag762-B22] Konishi S et al 2006. An SNP caused loss of seed shattering during rice domestication. Science. 312:1392–1396. 10.1126/science.1126410. 16614172

[kiag762-B23] Lee Y . 2019. More than cell wall hydrolysis: orchestration of cellular dynamics for organ separation. Curr Opin Plant Biol. 51:37–43. 10.1016/j.pbi.2019.03.009. 31030063

[kiag762-B24] Lee Y et al 2018. A lignin molecular brace controls precision processing of cell walls critical for surface integrity in Arabidopsis. Cell. 173:1468–1480.e9. 10.1016/j.cell.2018.03.060. 29731167

[kiag762-B25] Li C, Zhou A, Sang T. 2006. Rice domestication by reducing shattering. Science. 311:1936–1939. 10.1126/science.1123604. 16527928

[kiag762-B26] Li J, Su S. 2024. Abscission in plants: from mechanism to applications. Adv Biotechnol (Singapore). 2:27. 10.1007/s44307-024-00033-9. PMC11740850 39883313

[kiag762-B27] Lin Z et al 2012. Parallel domestication of the Shattering1 genes in cereals. Nat Genet. 44:720–724. 10.1038/ng.2281. PMC3532051 22581231

[kiag762-B28] Lionetti V . 2015. PECTOPLATE: the simultaneous phenotyping of pectin methylesterases, pectinases, and oligogalacturonides in plants during biotic stresses. Front Plant Sci. 6:331. 10.3389/fpls.2015.00331. PMC4429564 26029230

[kiag762-B29] Livak KJ, Schmittgen TD. 2001. Analysis of relative gene expression data using real-time quantitative PCR and the 2− ΔΔCT method. Methods. 25:402–408. 10.1006/meth.2001.1262. 11846609

[kiag762-B30] Lopato S et al 2006. Isolation of plant transcription factors using a modified yeast one-hybrid system. Plant Methods. 2:3. 10.1186/1746-4811-2-3. PMC1402289 16504065

[kiag762-B31] Ma X et al 2022a. Effect of roasting on the conformational structure and IgE binding of sesame allergens. J Agric Food Chem. 70:9442–9450. 10.1021/acs.jafc.2c01617. 35819315

[kiag762-B32] Ma X, Wang Z, Zheng C, Liu C. 2022b. A comprehensive review of bioactive compounds and processing technology of sesame seed. Oil Crop Sci. 7:88–94. 10.1016/j.ocsci.2022.05.003.

[kiag762-B33] Mao L et al 2000. JOINTLESS is a MADS-box gene controlling tomato flower abscission zone development. Nature. 406:910–913. 10.1038/35022611. 10972295

[kiag762-B34] Miao C et al 2018. Genotype-Corrector: improved genotype calls for genetic mapping in F2 and RIL populations. Sci Rep. 8:10088. 10.1038/s41598-018-28294-0. PMC6031647 29973633

[kiag762-B35] Micheli F . 2001. Pectin methylesterases: cell wall enzymes with important roles in plant physiology. Trends Plant Sci. 6:414–419. 10.1016/S1360-1385(01)02045-3. 11544130

[kiag762-B36] Mohnen D . 2008. Pectin structure and biosynthesis. Curr Opin Plant Biol. 11:266–277. 10.1016/j.pbi.2008.03.006. 18486536

[kiag762-B37] Nakano T et al 2012. MACROCALYX and JOINTLESS interact in the transcriptional regulation of tomato fruit abscission zone development. Plant Physiol. 158:439–450. 10.1104/pp.111.183731. PMC3252084 22106095

[kiag762-B38] Ning J et al 2023. The MYB transcription factor *Seed Shattering 11* controls seed shattering by repressing lignin synthesis in African rice. Plant Biotechnol J. 21:931–942. 10.1111/pbi.14004. PMC10106857 36610008

[kiag762-B39] Ogawa M, Kay P, Wilson S, Swain SM. 2009. Arabidopsis Dehiscence Zone Polygalacturonase1 (ADPG1), ADPG2, and QUARTET2 are polygalacturonases required for cell separation during reproductive development in Arabidopsis. Plant Cell. 21:216–233. 10.1105/tpc.108.063768. PMC2648098 19168715

[kiag762-B40] Pasternak T et al 2015. Protocol: an improved and universal procedure for whole-mount immunolocalization in plants. Plant Methods. 11:50. 10.1186/s13007-015-0094-2. PMC4625903 26516341

[kiag762-B41] Patterson SE . 2001. Cutting loose. Abscission and dehiscence in Arabidopsis. Plant Physiol. 126:494–500. 10.1104/pp.126.2.494. PMC1540116 11402180

[kiag762-B42] Pertea M et al 2015. StringTie enables improved reconstruction of a transcriptome from RNA-seq reads. Nat Biotechnol. 33:290–295. 10.1038/nbt.3122. PMC4643835 25690850

[kiag762-B43] Qi J et al 2017. Mechanical regulation of organ asymmetry in leaves. Nat Plants. 3:724–733. 10.1038/s41477-017-0008-6. 29150691

[kiag762-B44] Qureshi M, Langham DR, Lucas SJ, Uzun B, Yol E. 2022. Breeding history for shattering trait in sesame: classic to genomic approach. Mol Biol Rep. 49:7185–7194. 10.1007/s11033-022-07636-2. 35733066

[kiag762-B45] Roberts JA, Elliott KA, Gonzalez-Carranza ZH. 2002. Abscission, dehiscence, and other cell separation processes. Annu Rev Plant Biol. 53:131–158. 10.1146/annurev.arplant.53.092701.180236. 12221970

[kiag762-B46] Robinson MD, McCarthy DJ, Smyth GK. 2010. Edger: a Bioconductor package for differential expression analysis of digital gene expression data. Bioinformatics. 26:139–140. 10.1093/bioinformatics/btp616. PMC2796818 19910308

[kiag762-B47] Schmieder R, Edwards R. 2011. Quality control and preprocessing of metagenomic datasets. Bioinformatics. 27:863–864. 10.1093/bioinformatics/btr026. PMC3051327 21278185

[kiag762-B48] Sherman BT et al 2022. DAVID: a web server for functional enrichment analysis and functional annotation of gene lists (2021 update). Nucleic Acids Res. 50:W216–W221. 10.1093/nar/gkac194. PMC9252805 35325185

[kiag762-B49] Shimamura M . 2015. Whole-mount immunofluorescence staining of plant cells and tissues. In: Yeung E, Stasolla C, Sumner M, Huang B, editors. Plant microtechniques and protocols. Springer. p. 181–196.

[kiag762-B50] Su C . 2023. Pectin modifications at the symbiotic interface. New Phytol. 238:25–32. 10.1111/nph.18705. 36565041

[kiag762-B51] Teboul N, Magder A, Zilberberg M, Peleg Z. 2022. Elucidating the pleiotropic effects of sesame kanadi1 locus on leaf and capsule development. Plant J. 110:88–102. 10.1111/tpj.15655. 34964536

[kiag762-B52] Tran L-SP et al 2004. Isolation and functional analysis of Arabidopsis stress-inducible NAC transcription factors that bind to a drought-responsive cis-element in the early responsive to dehydration stress 1 promoter. Plant Cell. 16:2481–2498. 10.1105/tpc.104.022699. PMC520947 15319476

[kiag762-B53] Tripathy SK, Kar J, Sahu D. 2019. Advances in sesame (*Sesamum indicum* L.) breeding. In: Al-Khayri J, Jain S, Johnson D, editors. Advances in plant breeding strategies: industrial and food crops. Vol. 6: Springer. p. 577–635.

[kiag762-B54] Wormit A, Usadel B. 2018. The multifaceted role of pectin methylesterase inhibitors (PMEIs). Int J Mol Sci. 19:2878. 10.3390/ijms19102878. PMC6213510 30248977

[kiag762-B55] Wu H et al 2023a. Gibberellin signaling regulates lignin biosynthesis to modulate rice seed shattering. Plant Cell. 35:4383–4404. 10.1093/plcell/koad244. PMC10689197 37738159

[kiag762-B56] Wu H, He Q, Wang Q. 2023b. Advances in rice seed shattering. Int J Mol Sci. 24:8889. 10.3390/ijms24108889. PMC10219215 37240235

[kiag762-B57] Ye J et al 2018. WEGO 2.0: a web tool for analyzing and plotting GO annotations, 2018 update. Nucleic Acids Res. 46:W71–W75. 10.1093/nar/gky400. PMC6030983 29788377

[kiag762-B58] Yoon J, Cho L-H, Antt HW, Koh H-J, An G. 2017. KNOX protein OSH15 induces grain shattering by repressing lignin biosynthesis genes. Plant Physiol. 174:312–325. 10.1104/pp.17.00298. PMC5411160 28351912

[kiag762-B59] Yoon J et al 2014. The BEL 1-type homeobox gene SH 5 induces seed shattering by enhancing abscission-zone development and inhibiting lignin biosynthesis. Plant J. 79:717–728. 10.1111/tpj.12581. 24923192

[kiag762-B60] Yu Y, Kellogg EA. 2018. Inflorescence abscission zones in grasses: diversity and genetic regulation. Annu Plant Rev Online. 2:497–532. 10.1002/9781119312994.apr0619.

[kiag762-B61] Yun A, Kang J, Lee J, Song S-J, Hwang I. 2023. Design of an artificial transcriptional system for production of high levels of recombinant proteins in tobacco (*Nicotiana benthamiana*). Front Plant Sci. 14:1138089. 10.3389/fpls.2023.1138089. PMC9995837 36909433

[kiag762-B62] Zhang H, Langham DR, Miao H. 2021. Economic and academic importance of sesame. In: Miao H, Zhang H, Kole C, editors. The sesame genome. Springer. p. 1–18.

[kiag762-B63] Zhang H et al 2018. Identification of a SiCL1 gene controlling leaf curling and capsule indehiscence in sesame via cross-population association mapping and genomic variants screening. BMC Plant Biol. 18:296. 10.1186/s12870-018-1503-2. PMC6251216 30466401

[kiag762-B64] Zhao C, Avci U, Grant EH, Haigler CH, Beers EP. 2008. XND1, a member of the NAC domain family in *Arabidopsis thaliana*, negatively regulates lignocellulose synthesis and programmed cell death in xylem. Plant J. 53:425–436. 10.1111/j.1365-313X.2007.03350.x. 18069942

[kiag762-B65] Zhao C et al 2017. XYLEM NAC DOMAIN1, an angiosperm NAC transcription factor, inhibits xylem differentiation through conserved motifs that interact with RETINOBLASTOMA-RELATED. New Phytol. 216:76–89. 10.1111/nph.14704. 28742236

[kiag762-B66] Zhong R, Kandasamy MK, Ye Z-H. 2021. XND1 regulates secondary wall deposition in xylem vessels through the inhibition of VND functions. Plant Cell Physiol. 62:53–65. 10.1093/pcp/pcaa140. 33764471

