# Supplementary material for: Coordination of lignin and pectin remodeling by a NAC transcription factor regulates seed abscission in sesame (Sesamum indicum L.)
Source: Plant Physiol. 2026 Oct 14;202(2):kiag762. doi: 10.1093/plphys/kiag762 (PMC13711080; doi:10.1093/plphys/kiag762)
Supplement: Supplementary file 1 [file kiag762_supplementary_data.zip › Supplemetary figures_revised_2026-09-21.pdf]

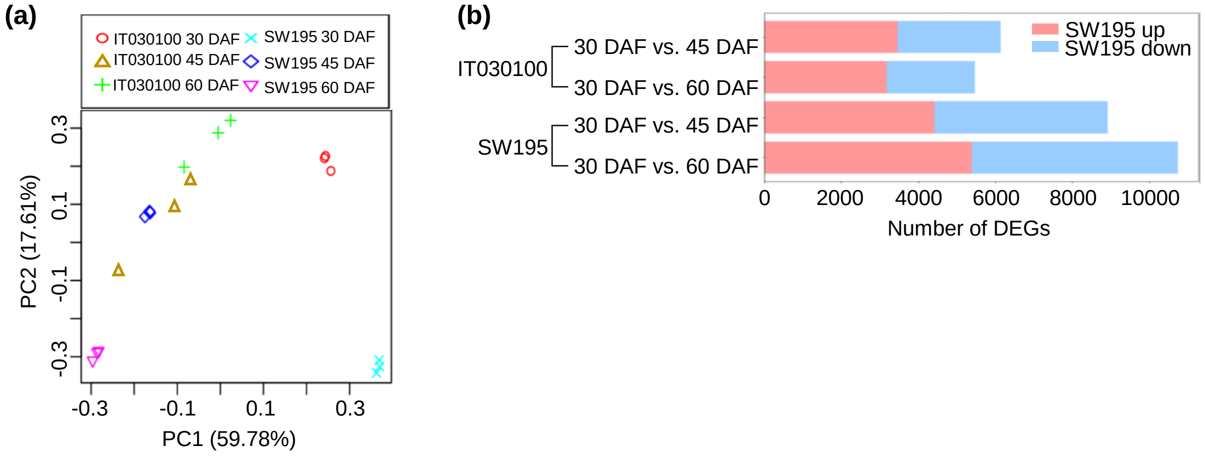

Figure S1. Differentially expressed genes (DEGs) between wild-type (IT030100) and SW195 sesame lines. a) Principal component analysis (PCA) of gene expression profiles in IT030100 and SW195 at the indicated day after flowering (DAF). Genes were considered to be significantly differentially expressed based on a false discovery rate (FDR) threshold of P < 0.05. **b)** Bar plot illustrating the number of genes exhibiting up- or down-regulation in SW195 at 45 and 60 DAF relative to 30 DAF. Upregulated genes are represented by red bars, while downregulated genes are depicted in blue.

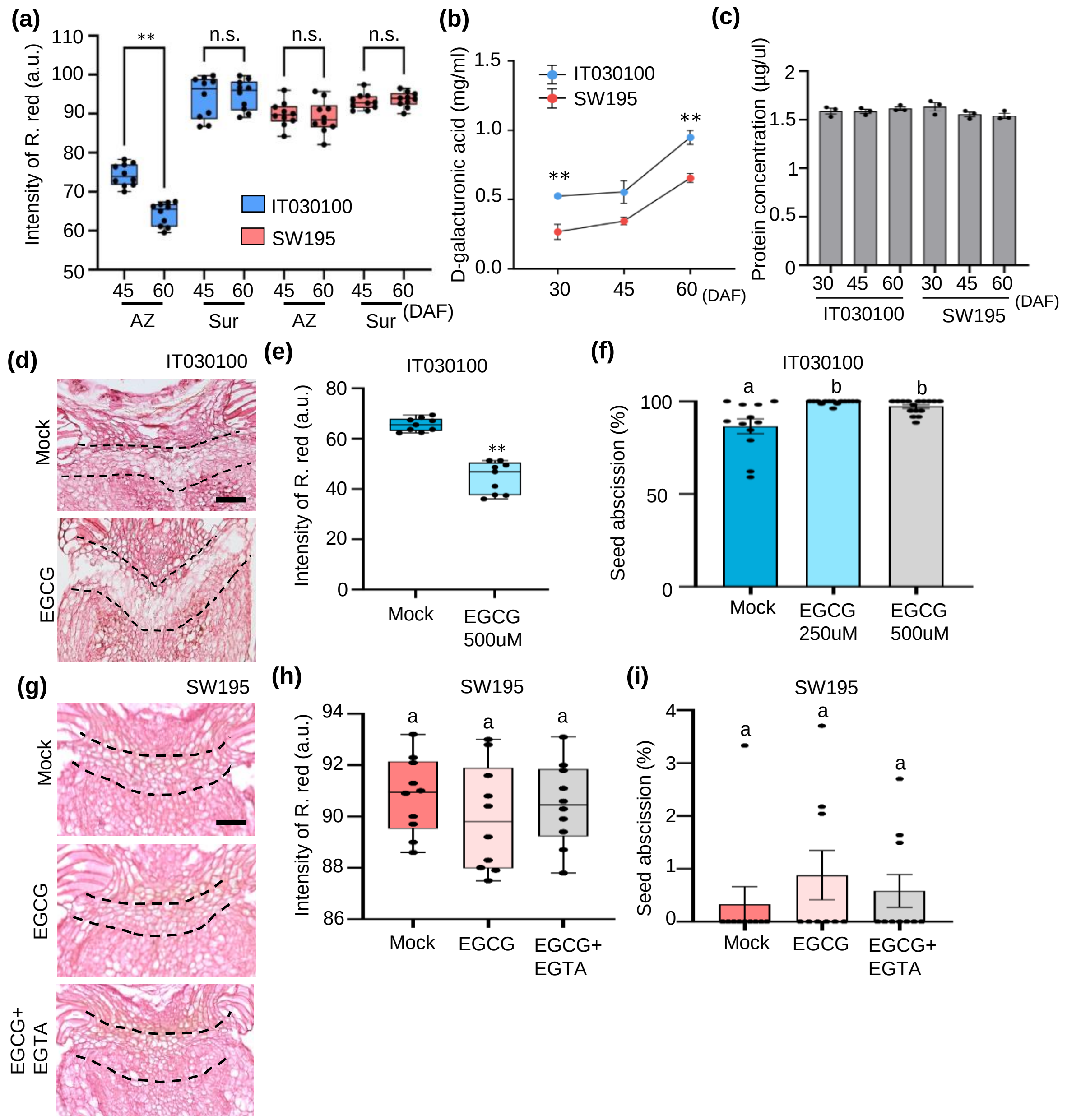

Figure S2. AZ-specific pectin cell wall remodeling in sesame plants.

a) Quantification of ruthenium red staining and a comparison of the intensity of AZ and surrounding tissue (Sur) as shown in Fig. 3B. *n* = 10 capsules. **b**) Polygalacturonase (PG) activity was measured using the 3,5-dinitrosalicylic acid (DNSA) method by quantifying the amount of D-galacturonic acid released after 6 h of incubation. Data represent mean ± SD of n = 3 independent biological replicates. **c)** Quantification of extracted protein concentration using Bradford protein assays, n = 3 biological replicates (in **Figure 3i, j**). **d-i)** Analysis of seed abscission following treatment with epigallocatechin gallate (EGCG) and ethyl glycol tetraacetic acid (EGTA) in IT030100 (**d-f**) and SW195 (**g-i**). Drugs were mixed with lanolin wax and placed on the petioles of 30 DAF capsules. 30 days after treatment, methyl-esterified pectin cell walls were visualized by ruthenium red staining (**d, g**) then quantified by measuring the intensity of staining in the AZ (**e, h**). *n* = 10 capsules. (**f, i**) Quantification of seed abscission phenotypes shown in (**d** and **g**, respectively). *n* = 10 capsules. All experiments were independently repeated at least three times. Error bars represent mean ± SD. **, P < 0.01; Student's t-test (**a, b, e**), Different letters indicate significant differences among group means. a.u., arbitrary units. P < 0.05, Kruskal–Wallis with Dunn's multiple comparison test (**f**) and one-way ANOVA with post hoc Tukey HSD test (**h, i**). n.s., not significant. Bar indicates 100μm.

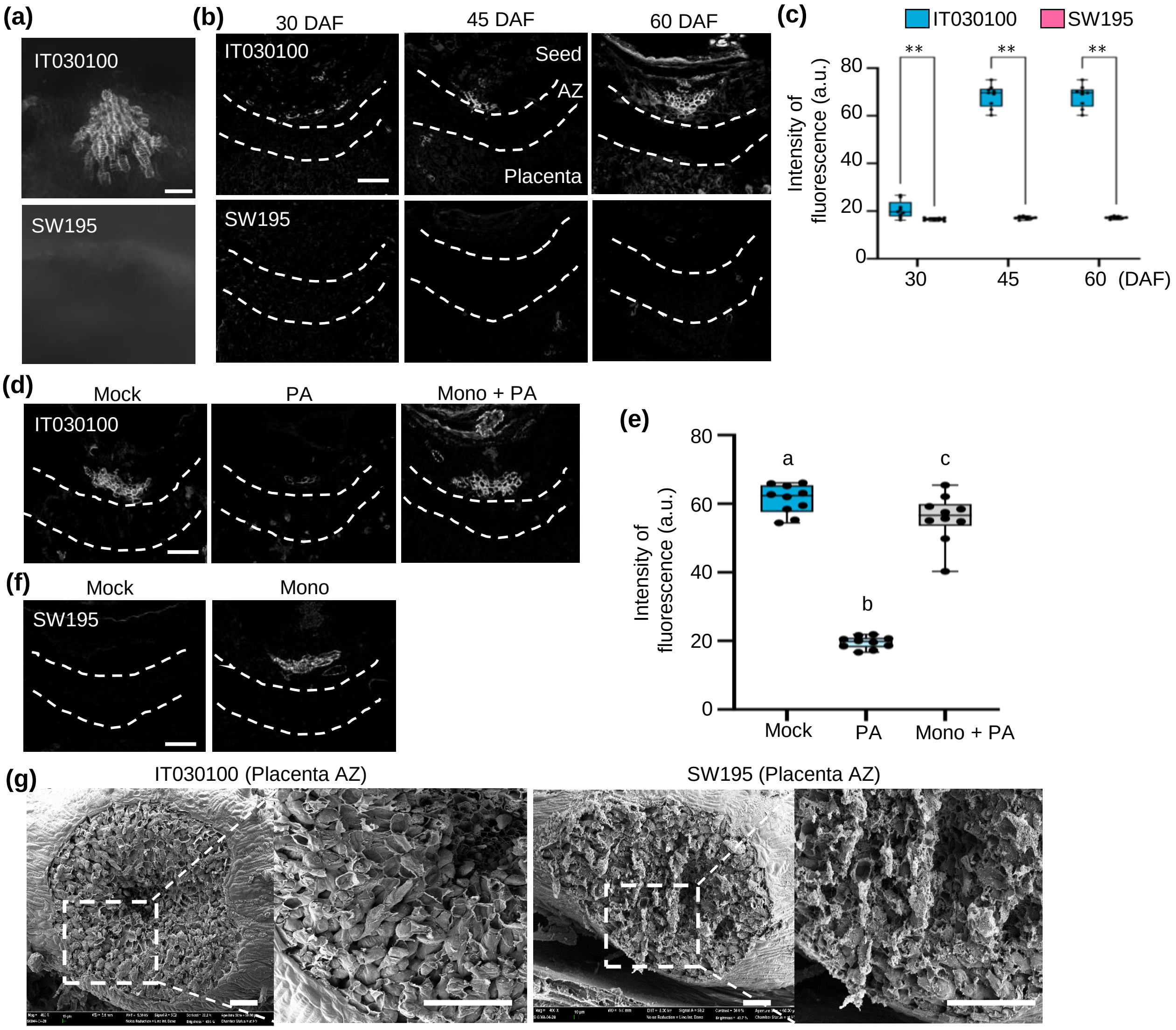

Figure S3. SEM analysis shows differences in seed separation phenotypes.
a, b) Lignin autofluorescence in whole-mounted seeds at 60 DAF (**a**) and paraffin sections of abscission zones (AZs) at the indicated stage (**b**) in IT030100 and SW195. **c**) Quantification of fluorescence in the AZs shown in **b**). *n* = 10 capsules. **d-f**) Lignin autofluorescence in the paraffin-sectioned AZs of IT030100 (**d**) and SW195 (**f**) treated with lanolin wax containing the indicated chemicals at 60 DAF. Drugs were mixed with lanolin wax then placed on the petioles of 30 DAF capsules for another 30 days. **e**) Quantification of fluorescence in the AZ shown in (**d**). *n* = 10 capsules. **g**) Scanning electron microscopy (SEM) images of the placenta show the separation surfaces in IT030100 and SW195 seeds at 60 DAF. All experiments were independently repeated at least three times. Error bars represent mean ± SD. **, P < 0.01; Student's t-test (**c**). Different letters indicate significant differences among group means. P < 0.05, Kruskal–Wallis with Dunn's multiple comparison test (**e**). Bars indicates 100μm.

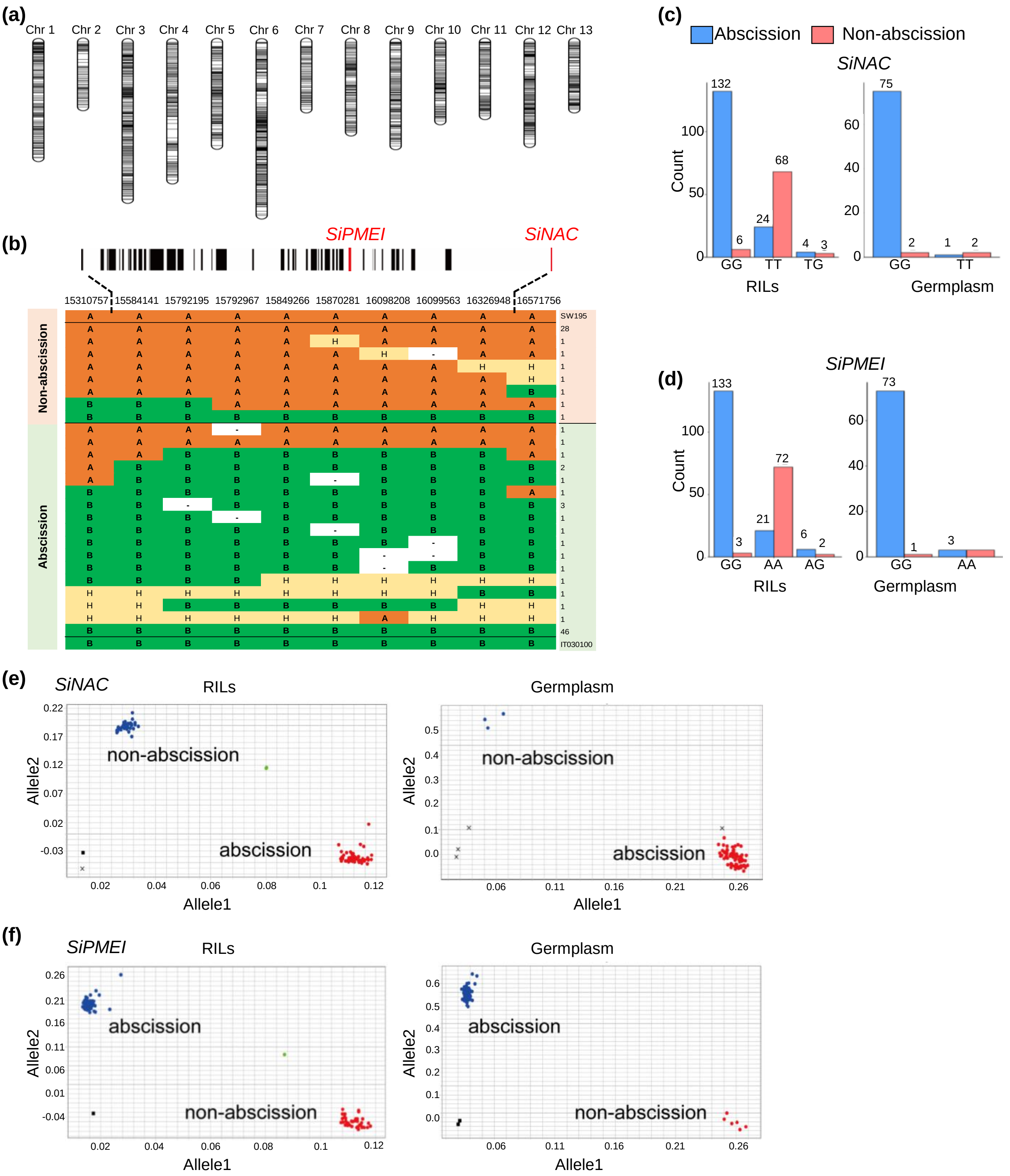

Figure S4. QTL analysis of non-seed abscission traits detected in sesame.
a) Genetic linkage maps of 13 chromosomes were constructed based on genotypic data from 100 recombinant inbred lines (RILs) and the two parental lines. **b)** Segregation of SNP haplotypes within the *qShattering9* locus in the RIL population, classified by phenotypes of non-abscission and abscission. Segregation of SNP haplotypes within the qShattering9 locus among RILs exhibiting non-abscission (score 1) or abscission (score 9) phenotypes in the manual-shaking assay (**Figure 5a**). Bars at the top of the figure represent the 44 protein-coding genes located within the *qShattering9* locus, with the candidate genes *SiPMEI* and *SiNAC* highlighted in red. A (orange background): SW195 allele; B (green background): IT030100 allele; H (yellow background): heterozygous allele. **c, d)** Number of abscission and non-abscission RILs and germplasms corresponding to the different alleles of the *SiNAC* (**c**) and *SiPMEI* (**d**) genes in sesame. **e, f)** Allele discrimination plot of the non-abscission candidate genes *SiNAC* (**e**) and *SiPMEI* (**f**) from a population of sesame RILs and germplasms.

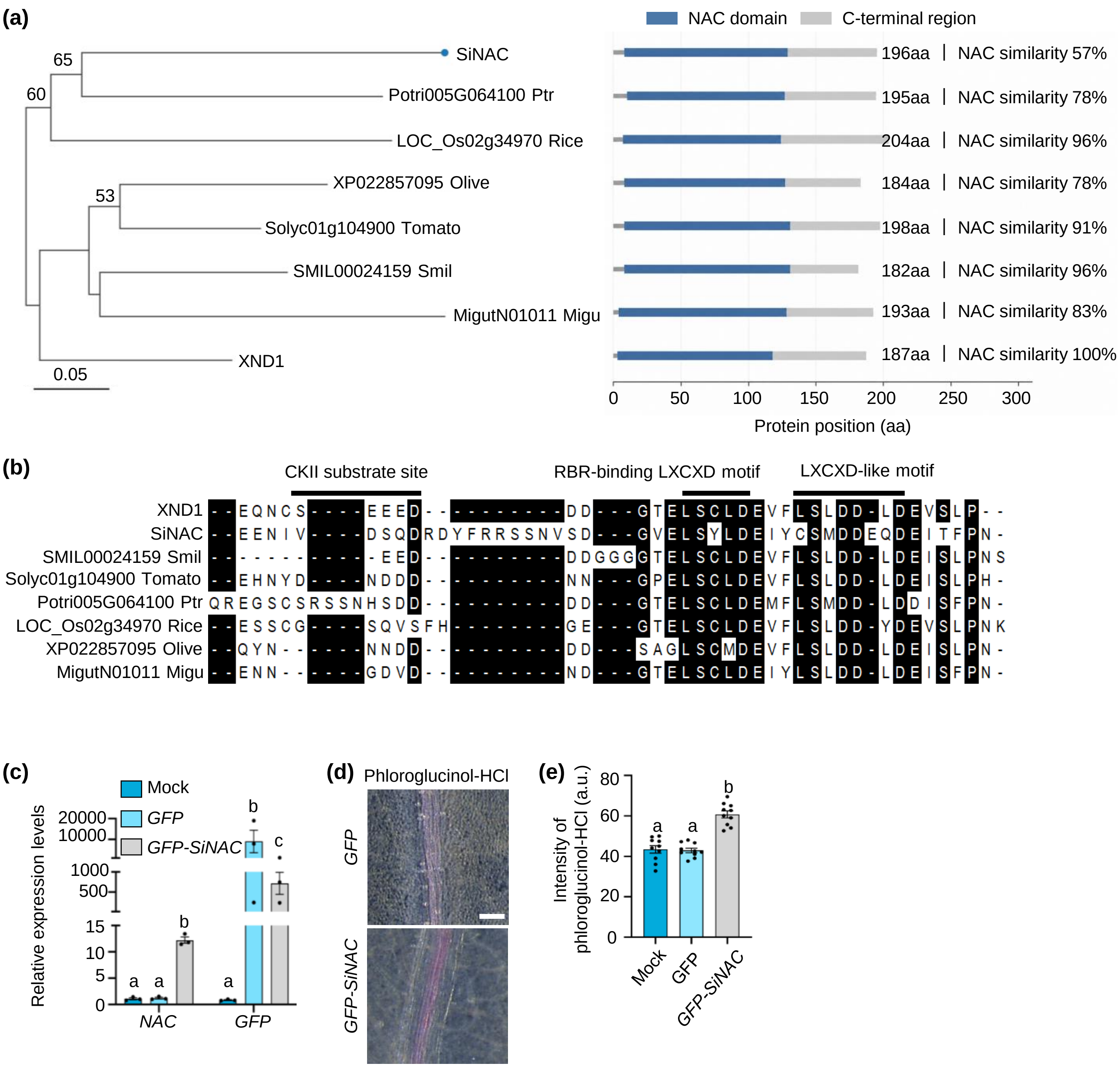

Figure S5. SiNAC promotes lignin accumulation and exhibits divergence in C-terminal motifs relative to XND1.

a) Phylogenetic analysis of SiNAC and homologs of Arabidopsis thaliana XND1 from the indicated plant species (left), and comparison of their NAC domains and C-terminal regions (right). Protein lengths and NAC-domain sequence similarities to A. thaliana XND1 are indicated. **b)** Multiple sequence alignment of the C-terminal regions of SiNAC and selected XND1 homologs. The CKII substrate residue, RBR-binding LXCXD motif, and LXCXD-mimic motif are indicated above the alignment. Conserved residues are highlighted in black. All proteins used for phylogenetic analysis are listed in **Supplemental Table S13. c)** Relative expression levels of *SiNAC* and *GFP* in agrobacterium-infiltrated sesame leaves in **Figure 6f,** as determined by RT-qPCR. 18S rRNA was used as the reference gene. Values represent the mean ± standard error of the mean (SEM) of three independent experiments. n = 3 biological replicates. **d)** Visualization of the lignin cell wall using phloroglucinol-HCl treatment in leaves in indicated plants. **e)** Quantification of lignin staining intensity in (**d**). *n* = 10 leaves. All experiments were independently repeated at least three times. Error bars represent mean ± SD. Different letters indicate significant differences between group means. The scale bar represents 0.05 amino acid substitutions per site **(a)**. P < 0.05, Kruskal–Wallis with Dunn's multiple comparison test (**c, e**). Bar indicates 200 µm **(d)**.

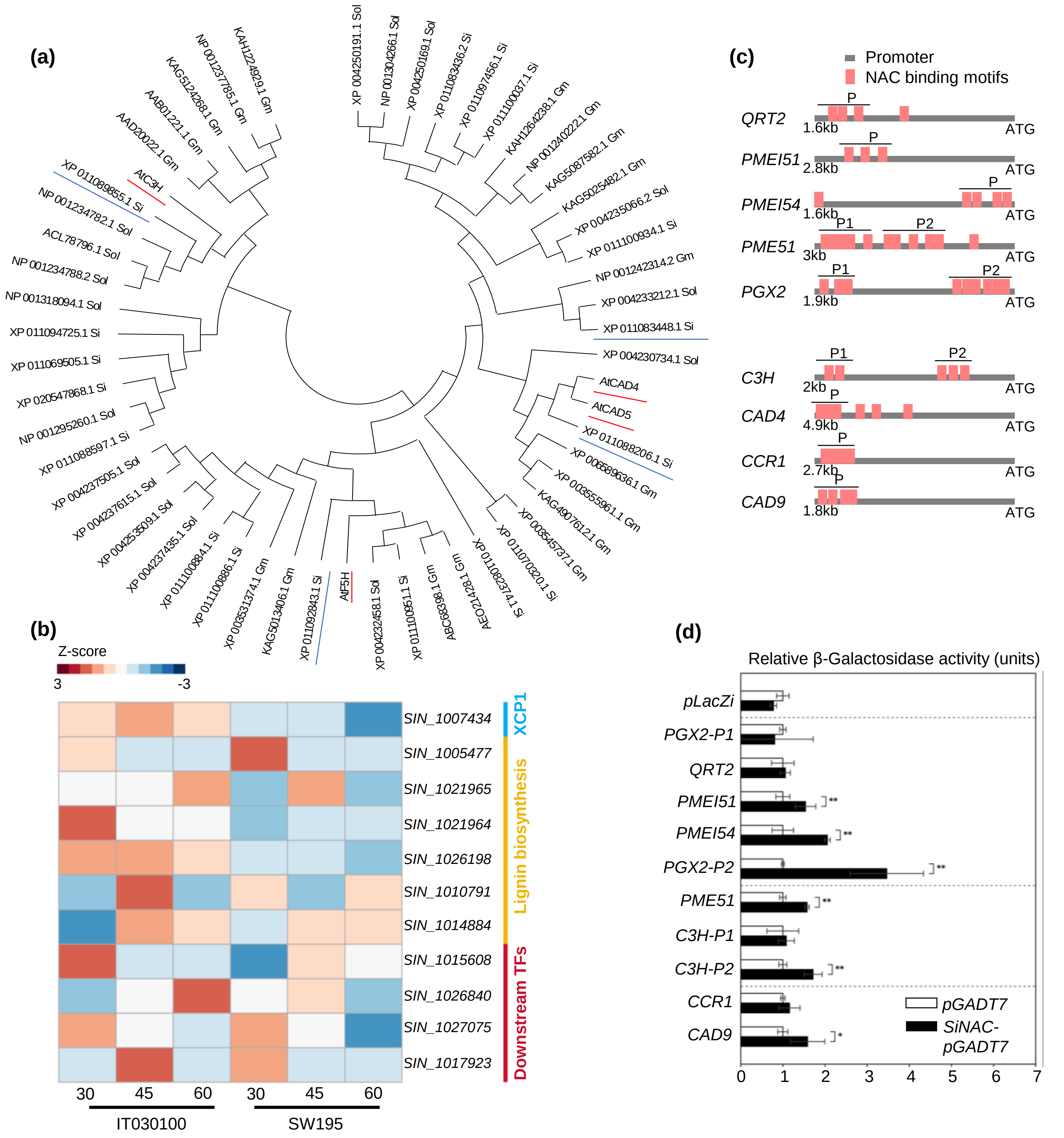

Figure S6. Phylogenetic, promoter, and expression analyses of cell wall-related genes associated with SiNAC and XND1.

a) Phylogram of lignin synthesis-related enzymes from different plant species. Amino acid sequences from different plant species were obtained from the NCBI database by BLASTP. Multiple protein alignment was then conducted using Clustal Omega, and this phylogenetic tree was constructed using the Neighbor-Joining method as implemented by MEGA X software. Proteins from Arabidopsis are shown in blue and proteins from sesame in red. All proteins used for phylogenetic analysis are listed in **Supplemental Table S14. b)** Heatmap showing expression profiles of sesame homologs of XND1-associated genes in IT030100 and SW195 at 30, 45, and 60 DAF. Genes are grouped as *XCP1*, lignin biosynthesis-related genes, and downstream transcription factors, as indicated. The color scale represents row Z-scores. See **Supplemental Table S15. c)** Schematic diagram of the promoter regions used for yeast one-hybrid (Y1H) assays. The gray lines represent the full-length promoter sequences, and red boxes indicate the positions of NAC binding domain. Horizontal lines denote the promoter fragments used in the Y1H assay. Numbers indicate the positions of nucleotides relative to the ATG start codon. P, P1, and P2 indicate the promoter fragments tested in each assay. **d)** Quantification of β-galactosidase activity using a chlorophenol red-β-D-galactopyranoside (CPRG) assay in Y1H experiments with the promoter fragments shown in **(c)**. n=6. The indicated reporter constructs were co-transformed with empty *pGADT7* (AD) or *SiNAC-pGADT7* (prey). All experiments were independently repeated at least three times.
